# Natural Products and Derivatives as Potential *Zika virus* Inhibitors: A Comprehensive Review

**DOI:** 10.3390/v15051211

**Published:** 2023-05-20

**Authors:** Rosângela Santos Pereira, Françoise Camila Pereira Santos, Priscilla Rodrigues Valadares Campana, Vivian Vasconcelos Costa, Rodrigo Maia de Pádua, Daniele G. Souza, Mauro Martins Teixeira, Fernão Castro Braga

**Affiliations:** 1 Department of Pharmaceutical Products, Faculty of Pharmacy, Universidade Federal de Minas Gerais, Belo Horizonte 31270-901, MG, Brazil; rosa.rspereira@gmail.com (R.S.P.);; 2 Department of Biochemistry and Immunology, Institute of Biological Sciences, Universidade Federal de Minas Gerais, Belo Horizonte 31270-901, MG, Brazil; 3 Department of Microbiology, Institute of Biological Sciences, Universidade Federal de Minas Gerais, Belo Horizonte 31270-901, MG, Brazil

**Keywords:** anti-ZIKV compounds, natural products, in silico assays, in vitro assays, in vivo assays

## Abstract

*Zika virus* (ZIKV) is an arbovirus whose infection in humans can lead to severe outcomes. This article reviews studies reporting the anti-ZIKV activity of natural products (NPs) and derivatives published from 1997 to 2022, which were carried out with NPs obtained from plants (82.4%) or semisynthetic/synthetic derivatives, fungi (3.1%), bacteria (7.6%), animals (1.2%) and marine organisms (1.9%) along with miscellaneous compounds (3.8%). Classes of NPs reported to present anti-ZIKV activity include polyphenols, triterpenes, alkaloids, and steroids, among others. The highest values of the selectivity index, the ratio between cytotoxicity and antiviral activity (SI = CC_50_/EC_50_), were reported for epigallocatechin gallate (SI ≥ 25,000) and anisomycin (SI ≥ 11,900) obtained from *Streptomyces* bacteria, dolastane (SI = 1246) isolated from the marine seaweed *Canistrocarpus cervicorni*, and the flavonol myricetin (SI ≥ 862). NPs mostly act at the stages of viral adsorption and internalization in addition to presenting virucidal effect. The data demonstrate the potential of NPs for developing new anti-ZIKV agents and highlight the lack of studies addressing their molecular mechanisms of action and pre-clinical studies of efficacy and safety in animal models. To the best of our knowledge, none of the active compounds has been submitted to clinical studies.

## 1. Introduction

*Zika virus* (ZIKV) and other arboviruses belonging to the *Flavivirus* genus (*Flaviviridae* family) are transmitted by mosquitoes, causing serious diseases in humans [1]. Some of them such as Zika, Dengue and Chikungunya viruses have spread globally and became major public health problems in tropical and subtropical regions of the globe [2].

ZIKV is genetically and serologically related to other flaviviruses such as Dengue, Yellow Fever, and West Nile viruses [3]. It is a positive-sense single-stranded RNA virus, presenting approximately 10.8 kb, consisting of two non-coding regions 3′ and 5′, three structural proteins (capsid, pre-membrane/membrane, and envelope) responsible for the structure of the viral particle, and seven non-structural proteins (NS1, NS2A, NS2B, NS3, NS4A, NS4B, and NS5) that participate in the replication and packaging of the viral genome [4,5].

The classic transmission of ZIKV occurs by the bite of mosquitoes of the genus *Aedes,* whereas transmission by blood transfusion, perinatal and through urine have been also described. ZIKV is the only member of the *Flavivirus* genus that is also transmitted by sexual and transplacental routes [4,6,7]. To date, there is no evidence of transmission by breastfeeding [8].

The first confirmation of ZIKV infection in Brazil dated from May 2015 [9]. Afterwards, the relationship between intrauterine infection by ZIKV and early microcephaly was established by physicians in the Brazilian northeast. Subsequently, ZIKV infection was associated with other severe neurological complications in babies born to ZIKV-infected mothers [10] and in ZIKV-infected adults [11]. The contact of pregnant women with ZIKV may result in fetuses and babies with brain calcifications, microcephaly and other congenital Zika syndromes (CZSs). Many authors suggest that even in babies considered normocephalic at birth, CZSs may cause complications such as delayed neurological development, loss of visual and auditory acuity, and difficulties in language proficiency [12,13,14]. In adults, one of the most important neurological manifestations is the Guillain–Barré syndrome, which is characterized by the peripheral neuropathy associated with progressive paralysis [15].

ZIKV continues to circulate in Brazil and in other countries with high potential for dissemination. Predictive models suggest that the geographic distribution of the *Aedes aegypti* mosquito, the main vector of ZIKV, continues to expand because of population growth and movement, uncontrolled urbanization, low prevention level, inadequate sanitary conditions, and climate changes [16]. Current actions to prevent infection by ZIKV encompass the use of barrier measures against the vector bite (use of repellents, mosquito nets, screens, long clothing), the elimination of possible breeding sites for larvae of *Aedes aegypti*, and the use of condoms to prevent sexual transmission of the virus [17,18].

To date, there is neither effective therapy against ZIKV infection and resulting complications such as microcephaly and Guillain–Barré syndrome nor vaccines for its prevention; therefore, the World Health Organization (WHO) considers urgent the research and development of new drugs against this virus [19,20]. More accurate diagnostic tests are also demanded, since ZIKV infection is difficult to diagnose and to differentiate clinically from Dengue, Chikungunya, and other viral infections [21].

The research of natural products (NPs) has been proved to be a valid strategy for developing important drugs currently used in therapy, and they also play an important role in the discovery of new antiviral agents [22,23,24,25,26,27]. In this context, the search for compounds active against ZIKV is relevant and may result in the development of new antivirus drugs to be adopted for the prevention and treatment of future generations. Several classes of NPs have been described in the literature as active against ZIKV in different experimental models, including polyphenols, triterpenes, alkaloids, and steroids, among others. Therefore, the goal of the present work was to undertake a comprehensive revision on NPs that have been reported to exhibit potential effect against ZIKV by using predictive computational tools, as well as by in vitro and in vivo models of viral infection, along with their mechanisms of antiviral activity.

## 2. Methodology

A literature review was conducted on NPs and derivatives tested against ZIKV. The following databases were accessed: PubMed/MEDLINE, Scientific Electronic Library Online (SciELO), Scopus, Google Scholar, Web of Science, Science Direct, SciFinder and *Literatura Latino-Americana e do Caribe em Ciências da Saúde* (LILACS). Articles written in English and published up to December 2022 were reviewed. Indexed keywords from Medical Subject Headings (MeSH) and *Descritores em Ciências da Saúde* (DeCS) were used to build up search strategies, and the following ones were adopted: “Zika virus” combined with the descriptors “medicinal plants”, “plants”, “bioactive compound”, “antiviral bioactive compounds”, “phenolic compounds”, “flavonoids”, “alkaloids”, “terpenoids”, “antiviral natural products”, “antiviral activity”, “biological product”, “natural products”, “inhibitors”, “activity”, “antiviral effect”, “viral life cycle”, “compounds”. The “AND” connector was used between the terms as, for example, “Zika virus” and “medicinal plants”. The search results were saved and compiled using Microsoft Excel version 2016 software to select potential eligible studies.

The revision was focused on scientific articles reporting NPs from plants, animals, fungi, and bacteria with antiviral action against ZIKV assayed by in silico, in vitro, and in vivo models. Reports on synthetic and semisynthetic derivatives of NPs were also selected for revision, whereas review articles, notes, editorials, abstracts presented at scientific meetings, experience reports, dissertations and theses were excluded. The above-mentioned inclusion and exclusion criteria were strictly followed for selecting eligible articles for revision. The title, abstract, and keywords were firstly read to pre-select the articles of interest, which were carefully browsed subsequently for inclusion in this paper. The number of papers reporting NPs tested against ZIKV published between January 1997 and December 2022 is presented in Figure S1 as Supplementary Material.

## 3. NPs with Potential Anti-ZIKV Activity

**Figure 1:**
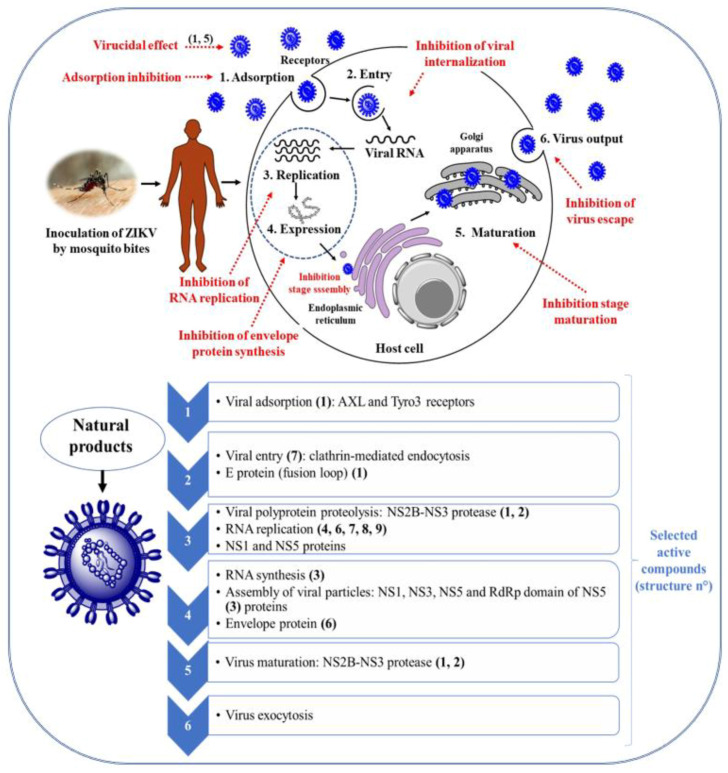
Molecular targets of *Zika virus* reached by natural products (NP) and possible outcomes on the viral life cycle. Dotted arrows indicate targets of NPs against the virus. In silico targets: NS1, NS2B-NS3 protease, NS3, NS5 methyltransferase, RdRp domain of NS5 protein; In vitro targets: stages of virus adsorption and internalization, virucidal effect; In vivo targets: RNA replication and RNA synthesis. Examples of NPs active in some of these targets: (**1**) (-)-epigallocatechin gallate; (**2**) myricetin; (**3**) fenretinide; (**4**) 25-hydroxycholesterol; (**5**) dolastane; (**6**) lycorine; (**7**) chloroquine; (**8**) ascomycin; (**9**) anisomycin. The chemical structures of these NPs are depicted in Figure 2.

In general, the action NPs and derivatives on the reproduction cycle of ZIKV involves virucidal effect, i.e., action on ZIKV outside the host cell, inhibition of virus adsorption, inhibition of viral internalization, blockade of viral envelope and protein synthesis, inhibition of viral RNA replication, and inhibition of the release of the virus from the host cell (Figure 1).

The potential anti-ZIKV activity of different classes of NPs has been investigated by using in silico, in vitro and in vivo assays, and the reported results are discussed sequentially in Section 3.1, Section 3.2 and Section 3.3.

### 3.1. NPs with Potential Anti-ZIKV Activity Identified by In Silico Tools

The ability of NPs to interact with molecular targets of ZIKV has been assessed by using in silico tools, mainly molecular docking, and the main targets so far identified and its possible effects on the viral life cycle are depicted in Figure 1. In general, literature data point out polyphenols and alkaloids as the best molecular docking ligands for ZIKV target proteins with high affinity for protein receptors. The literature data are presented in the following subsections based on the assayed target protein. Table S1 of the Supplementary Material summarizes NPs that interact with the relevant molecular targets of ZIKV, which is disclosed by in silico assays.

#### 3.1.1. ZIKV NS2B-NS3 Protease and NS3 Proteins

Several alkaloids have been reported to act as inhibitors of the ZIKV NS2B-NS3 protease, including bromocriptine (a derivative of the alkaloid ergoline) [28], hydroxychloroquine (a semisynthetic derivative of quinine) [29], and the bis-indole alkaloids flinderol A and flinderol B [30,31]. Seven novel berberine derivatives were tested as possible inhibitors of the ZIKV NS2B-NS3 protein, and one presented a high value of binding energy [32]. Additionally, the isoquinoline alkaloid cassiarin D, along with the flavonoids 3′-*O*-methyldiplacone and exiguaflavanone A, in addition to the sesquiterpene lactucopicrin, showed strong anchoring properties with the NS3 helicase of ZIKV [33].

Roy and coworkers [34] reported the flavonoids myricetin, quercetin [35], isorhamnetin, luteolin, and apigenin, along with curcumin, compounds commonly found in different vegetables and fruits, as non-competitive allosteric inhibitors of the ZIKV NS2B-NS3 protease [36]. Interestingly, curcumin also showed the strongest binding with tumor necrosis factor (TNF) in a molecular docking study [36]. Thus, TNF inhibition can reduce the inflammatory process caused by ZIKV infection, decreasing symptoms and the risk of future complications. Another flavanone, naringenin, was able to interact with the NS2B-NS3 protease protein domain of ZIKV, acting as a non-competitive inhibitor [37].

The flavanone hesperidin exhibited significant in silico inhibition of ZIKV NS2B-NS3 protease, which is important in the ZIKV replication process [38], along with the chalcones angusticornin B and curaridin [30,31]. More recently, a set of 150 NPs and semisynthetic derivatives was subjected to the molecular docking against the ZIKV NS2B-NS3 protease. Quercetin, rutin, and pedalitin, isolated from *Pterogyne nitens*, presented the best affinity to this ZIKV protease [39].

A total of 38 flavonoids was subjected to molecular docking and molecular dynamics simulation studies against the ZIKV NS2B-NS3 protease [40]. According to the authors, epigallocatechin gallate, epigallocatechin gallate-7-*O*-glucopyranoside, epigallocatechin gallate-4’-*O*-α-glucopyranoside, isoquercetin, rutin, and sanggenon O were capable of interacting with the catalytic triad (His51, Asp75 and Ser135) from the active site of the ZIKV NS2B-NS3 protease. These compounds showed stability in the active site of the protein–ligand complex [40]. Additionally, a virtual screening of 5550 NPs and derivatives disclosed baicalein, catechin, muricatetrocin, canthin, eleutheroside B, ellagic acid, epigallocatechin, neoandrographolide, ponapensin, and sangennon as capable of establishing hydrogen bonding interaction with amino acids of the ZIKV NS3 protein helicase [41].

Other classes of NPs have been also reported as potential inhibitors of the ZIKV NS2B-NS3 protease. Hence, the diterpenes bisandrographolide, andrographolide, and andrographiside, isolated from *Andrographis paniculate* showed appropriate interactions at the binding site of the above-mentioned protease [42]. Novobiocin, isolated from species of *Streptomyces* bacteria, interacted with the ZIKV NS2B-NS3 protease with high stability [43], whereas glycyrrhetinic acid and its derivatives 3-*O*-acetyl-30-aminopyridine and 1,2,3-thiadiazole-30-butyl interact with the ZIKV NS2B-NS3 protease through hydrogen bonds [44].

#### 3.1.2. ZIKV NS5 Protein

A total of 2035 plant compounds comprising alkaloids (322), terpenoids (113), chalcones (101), flavonoids (483), lignans (211), carboxylic acids (255), polyphenols (301), and quinones (249) was screened against the ZIKV-NS5 protein, focusing on the domains of methyltransferase (MTase) and RNA-dependent RNA polymerase (RdRp). Overall, 13 compounds strongly inhibited the ZIKV-NS5 protein in the Mtase domain, whereas 17 compounds significantly inhibited its RdRp domain. These 30 compounds were further analyzed and validated for their reactivity in the binding pocket of the ZIKV-NS5 protein, i.e., in the conformation of the ligand-NS5 domain. Kercetagetin and ferulic acid were found to be the most active compounds within the ZIKV-NS5 binding pocket [45].

For ZIKV NS5 methyltransferase, significant docking properties were exhibited by cimicifenol, cimiracemate B and rosmarinic acid, the chalcones kanzonol Y and curaridin, the aurone kanzonol V, the flavone solophenol D, and the lignan (-)-asarinin. Furthermore, anchoring properties for ZIKV NS5 RNA-dependent RNA polymerase were demonstrated for 4’,7-digaloylcatechin di-*O*-dimethylsoguaiacin and 2,4,4’-trihydroxy-3,3′-diprenylchalcone in addition to the alkaloid flinderol B [28]. In its turn, the flavones baicalein and baicalin exhibited the strongest binding affinities for the ZIKV NS5 protein, while the ZIKV envelope protein was the lowest interaction target of these compounds [46].

Thirty-five compounds inhibited the ZIKV-NS5 protein in the MTase domain, of which the flavonoids silybin B and silybin C exhibited the highest affinity energy (−10.2 kcal/mol). Additionally, 29 compounds inhibited the ZIKV-NS5 protein in the RNA-dependent RNA polymerase (RdRp) domain, the highest affinity energy being found for the flavonoid isopomiferin (−9.2 kcal/mol). This compound was also identified as a potential inhibitor of both NS5 MTase and NS5 RdRp of ZIKV in addition to four dengue serotypes proteins [47]. Moreover, polydatin, dihydrogenistin, liquiritin, rhapontin, and cichoriin were successfully bound inside the pocket of ZIKV NS5 RdRp [48].

Sinefungin, an adenosine derivative originally isolated from the bacteria *Streptomyces griseoleus*, was characterized as a competitive inhibitor of ZIKV metiltransferase [49]. In its turn, lycorine was shown to interact specifically with the NS5 RdRp domain of the protein [50].

A set of 72 compounds from *Piper nigrum* and *Salvia rosmarinus* was tested against ZIKV. Among them, 26 constituents of *P. nigrum* and 31 compounds of *S. rosmarinus* showed effective drug-likeness properties. Piperine and isoscutellarein have shown remarkable inhibitory potential against the ZIKV NS5 RdRp protein [51]. In its turn, the polyphenol theaflavin, isolated from *P. nigrum*, interacts with ZIKV NS5-MTase by hydrogen bounds [52].

#### 3.1.3. ZIKV Envelope Protein

Twenty-five compounds comprising flavones, alkaloids, and polyphenols found in species of Asteraceae, Acanthaceae, Combretaceae, Lamiaceae, Phyllanthaceae and Polygonaceae were selected for virtual screening against the ZIKV envelope protein. Tannic acid isolated from *Terminalia arjuna* (Combretaceae) showed better interaction with the ZIKV envelope E protein [53], and the authors suggested that it is potentially a good inhibitor of virus adsorption and could be further investigated to control ZIKV infection.

Epigallocatechin gallate showed high affinity for the ZIKV E protein (dimers I and III), preventing the conformational change of dimer III, which is required for ZIKV entry into the host cells [54]. Another study reported epicatechin to present good docking scores with the domain III of the envelope protein along with baicalin, isonimolicinolide, madecassic acid, and apigenin-7-*O*-beta-D-glucopyranoside [41]. In the same direction, pentagalloylglucose, parishin A, and stevioside showed remarkable docking scores with the ZIKV envelope (E) protein that represents the major target for neutralizing antibodies [55].

The protoberberine alkaloid palmatine and the alkaloid harringtonine, found in *Cephalotaxus* species, have been described to bind satisfactorily to the ZIKV envelope protein [30,31]. In its turn, the constituents of *Epiphyllum oxypetalum* leaves 4-hydroxy-2-methylacetophenone, stigmasterol, 6-octen-1-ol,3,7-dimethy-megastigmatrienone, myclohexylmethyl hexyl ester, and testerone cypionate showed high affinity for the ZIKV E protein through hydrogen bonds and Van der Waals forces interactions [56].

#### 3.1.4. ZIKV Multitarget Assays

Some NPs may act on different molecular targets of ZIKV, and this feature has been explored in silico. Hence, 4 out of 44 flavonoids found in *Azadirachta indica—*rutin, nicotiflorin, isoquercitrin, and hyperoside—showed better docking scores against the NS2B-NS3 protease and NS5 RNA-dependent RNA polymerase [57]. In its turn, the chalcones 4-hydroxyderricin, xanthoangelol, and xanthoangelol-E, isolated from *Angelica keiskei*, were able to inhibit in silico the ZIKV NS2B-NS3 protease and NS5 polymerase [58]. Furthermore, the apocarotenoid bixin, obtained from *Bixa orellana*, along with its derivatives annatto, crocetin dimethyl ester, ethyl bixin, mycorradicin, norbixin, and transcrocetin were reported to interact with NS2B-NS3 and NS5 proteases [59]. Molecular docking performed between β-caryophyllene, the major constituent of *Lippia alba* essential oil, and ZIKV targets NS1, NS2B-NS3, and NS5 disclosed better affinity with the NS2B-NS3 protein complex and with the NS5 protein [60].

The bioactive metabolites 5,3′-dihydroxy-3,6,7,8,4′-pentamethoxyflavone, 5-hydroxy-3,6,7,8,3′,4′-hexamethoxyflavone, and myricetin-3-*O*-rhamnoside from *Marcetia taxifolia* showed better interactions with the ZIKV-NS3-helicase than ZIKV-NS5-RNA-polymerase [61]. Galloylquinic acid, isolated from *Euphorbia hirta*, along with bacopaside III and bacopaside A, obtained from *Bacopa monnieri*, were reported to interact with NS1, NS3 and the envelope protein domain III of ZIKV [62].

Compounds found in the aqueous extracts of *Momordica charantia* (saikosaponin D and benzoyl paeniflorin), *Vitex negundo* (macrophylloside D), and *Blumea balsamifera* (cyanidin 3-5-diglucoside) were subjected to docking studies against several ZIKV targets: helicase, protease, methyltransferase, RNA-dependent RNA polymerase, and envelope protein [63]. These compounds exhibit higher binding affinity to the viral replication proteins than to the envelope protein.

Resveratrol exhibited strong binding affinity for ZIKV NS5 RNA RdRp protein as well as for the cellular targets cytochrome P450 family 1, subfamily B, polypeptide 1 (CYP1B1), aryl hydrocarbon receptor (AHR), dihydroorotate dehydrogenase (DHODH), and guanosine monophosphate reductase 2 (GMPR2) [64]. In its turn, chicoric acid, luteone, reserpine, and rosmarinic acid showed better biding affinity with the NS2B-NS3 protease, envelope protein, capsid protein, and NS5 RNA-dependent RNA polymerase protein [65].

Tangeretin is a polymethoxyflavone from the peel oil of *Citrus reticulata*. This compound was able to interact with the ZIKV NS1 protein by establishing strong hydrogen bonds and with ZIKV NS5 [66].

The prediction results from the in silico studies herein reviewed demonstrate the potential activity of NPs against molecular targets of ZIKV. The judicious validation of compounds exhibiting the best docking scores, through in vitro and in vivo assays, might disclose promising compounds for developing new drugs to fight ZIKV infection and minimize ZIKV-mediated pathologies [67]. Computational approaches generate activity predictions that can contribute to accelerate the development of new active compounds in addition to predictions of pharmacokinetics and the safety of anti-ZIKV compounds [68]. However, data generated by in silico assays must be carefully analyzed, keeping in mind the need to correlate them with in vitro and in vivo test results.

### 3.2. Extracts, NP, and NP-Derivatives Active In Vitro against ZIKV

**Figure 2:**
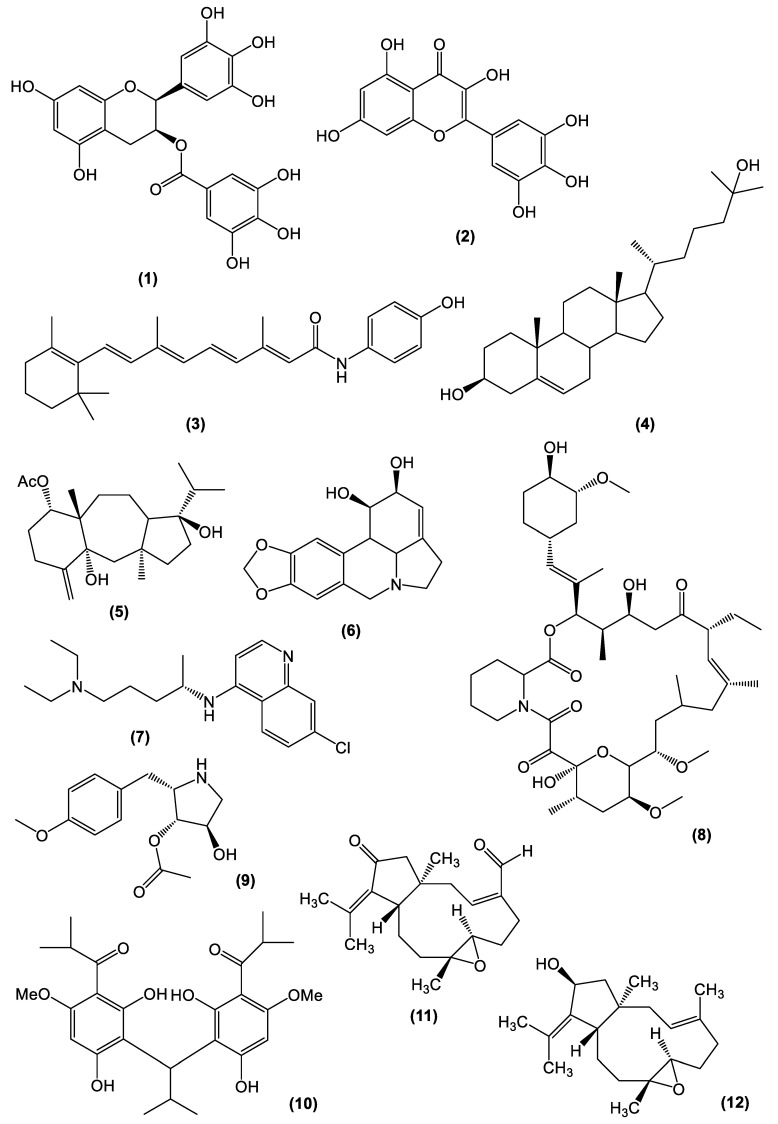
Chemical structures of selected NPs with promising anti-*Zika* virus activity (selectivity index >500 and antiviral activity demonstrated in more than one assay: in silico, in vitro or in vivo). (**1**) (-)-Epigallocatechin gallate, (**2**) myricetin, (**3**) fenretinide, (**4**) 25-hydroxycholesterol, (**5**) dolastane, (**6**) lycorine, (**7**) chloroquine, (**8**) ascomycin, (**9**) anisomycin, (**10**) Eucalyprobusone G, (**11**) (1*R*, 3Z, 7*R*, 8*R*, 11*S*)-7,8-epoxy-13-ketodolabell-3-en-16-al, (**12**) (1*R*, 7*R*, 8*R*, 11*S*, 13*S*)-7,8-epoxy-13-hydroxy-dolabella-3,12(18)-diene.

The selectivity index (SI) is the ratio that measures the window between cytotoxicity and antiviral activity. Thus, SI is the ratio of median cytotoxic concentration (CC_50_) to median effective concentration (EC_50_), which is related to a specific biological effect: in this case, the activity against ZIKV. Theoretically, a high SI ratio would indicate an effective and safe compound for the in vivo treatment of a certain virus infection. An SI value ≥ 10 is recommended for selecting a compound/extract potentially useful to be further investigated as an antivirus agent [69]. Therefore, we included in this review mostly samples that presented SI values ≥ 10, comprising isolated compounds from natural sources, derivatives, and some plant extracts, as described in this section. On the other hand, data for samples with SI below the adopted cutoff value are reported on Table S2 as Supplementary Material. The SI values of several samples are being described in the published data; for some of them, there are only EC_50_ values reported, whereas for others, only the percentage of inhibition is informed. These data are compiled in Table 1, along with the cell lineage used in the assays, and the antivirus mechanism of action, when available. The chemical structures of some selected compounds, which elicited potent anti-ZIKV activity in vitro and/or in vivo, are depicted in Figure 2.

#### 3.2.1. Plant Extracts

Extracts obtained using solvents of different polarity and essential oils from plant species have already been tested in vitro against ZIKV, and a wide range of SI values has been reported. Hence, the aqueous extract of the aerial parts of *Aphloia theiformis* showed anti-ZIKV effect and was not cytotoxic (CC_50_ = 3000 μg/mL) to Vero cells. The concentration-dependent activity of *A. theiformis* extract was observed through a 2-log reduction in viral load recovered from ZIKV African strain MR766 in comparison with the viral inoculum (EC_50_ of 100 μg/mL, SI = 30). In Vero cells, this extract (500 μg/mL) reduced the infectivity of the ZIKV Asian PF13 strain at 2-log viral load; and for the Brazilian ZIKV strain (ZV BR 2015/15261), the extract reduced 3-log viral load in Huh7.5 cells. The *A. theiformis* extract inhibited ZIKV adsorption in Vero cells and also showed antiviral effect against *Dengue virus* (DENV) [70].

The ethanolic extract of the seaweed *Osmundaria obtusiloba* exhibited low cytotoxicity in Vero cells, inhibited ZIKV replication in a concentration-dependent manner (CC_50_ = 525.00 ± 3.11 μg/mL, EC_50_ = 1.82 ± 0.49 μg/mL, SI = 288) and showed virucidal effect. The effect of *O. obtusiloba* extract (0.5 µg/mL) against ZIKV increased significantly with the addition of ribavirin (0.5 µM), a synthetic antiviral analog of guanosine, due to a synergistic effect [71].

The aqueous extract of *Psiloxylon mauritianum* aerial parts, tested at 100 μg/mL, inhibited ZIKV infection in Vero cells, decreasing by 2-log the production of infectious viral particles from African (MR766) and Asian (PF13) strains [72]. This extract maintained 90% viability of Vero and A549 cells and did not cause DNA damage to A549 cells even at cytotoxic concentration (500 µg/mL), which were determined using MTT and COMET methods, respectively. The obtained results in Vero cells (CC_50_ = 1044.00 ± 106.20 µg/mL, EC_50_ = 19.50 ± 4.80 µg/mL and SI = 53.5) showed that the antiviral activity of *P. mauritianum* extract significantly affects the attachment of ZIKV to the cell surface and also that its action is mediated by an interaction between the extract and the ZIKV particles. The extract was also active against four dengue serotypes [72]. It is important to highlight that the *P. mauritianum* extract is rich in phenolic compounds, such as gallic acid, quercetin and kaempferol.

The ethanolic extract of *Himatanthus bracteatus* showed low cytotoxicity in Vero cells (CC_50_ = 348.02 ± 5.75 μg/mL) and significant activity against ZIKV, yellow fever virus and *Dengue virus* sorotype 2 (DENV-2) (EC_50_ = 31.46 ± 0.72, 17.43 ± 3.90 and 25.03 ± 1.20 μg/mL, respectively) with SI > 10, whereas the *n*-hexanic extract was inactive against the assayed viruses (SILVA et al., 2019) [73]. The sesquiterpene glycoside plumieride, the major constituent of *H. bracteatus*, was active against ZIKV (EC_50_ = 14.49 ± 0.72 μg/mL and SI = 15.97) [73].

The essential oil from the aerial parts of *Ayapana triplinervis* was not cytotoxic for A549 cells and showed anti-ZIKV activity (EC_50_ = 38.0 μg/mL, SI = 12.5). Dimethylthio-hydroquinone ether, isolated from the essential oil of *A. triplinervis*, also showed antiviral activity against ZIKV (EC_50_ = 45.0 μg/mL, SI = 9.1). The inhibition of ZIKV mediated by dimethylthio-hydroquinone ether occurs after the virus binds to the cell membrane and is explained by the inability of the ZIKV-bound particles to be internalized in the host cell [74].

The chloroform and ethyl acetate extracts from the seaweed *Dictyota menstrualis*, tested at 20 µg/mL, inhibited ZIKV replication in Vero cells by 42% and 38%, respectively. Fractionation afforded the active F-6 fraction derived from the chloroform extract (CC_50_ = 592.00 µg/mL, EC_50_ = 2.80 ± 0.10 µg/mL, SI = 211.43) and FAc-2 fraction derived from the ethyl acetate extract (CC_50_ = 482.00 µg/mL, EC_50_ = 0.81 ± 0.04 µg/mL, SI = 595.06). Regarding the mechanism of action, the FAc-2 fraction showed virucidal potential, and the F-6 fraction inhibited viral adsorption. The combination of FAc-2 with ribavirin (0.25 µg/mL of FAc-2 and 0.25 µM of ribavirin) elicited a strong synergistic effect that inhibited ZIKV replication completely [75]. The chemistry of *D. menstrualis* has been investigated and resulted in the isolation of α,β-unsaturated dialdehyde diterpenes such as 6-hydroxy-dichotoma-2,13-diene-16,17-dial (xeniane skeleton), 6-acetoxydichotoma-2,13-diene-16,17-dial (dichotomane skeleton), 5-hydroxy-1,6-cycloxenia-2,13-diene-16,17-dial and 5-acetoxy-1,6-cycloxenia-2,13-diene-16,17-dial (cycloxeniane skeleton), and acetoxydictyodial (xeniane skeleton) [75].

The methanolic extract of *Psychotria viridis* leaves induced anti-ZIKV activity in Vero cells with SI > 28.85. The extract showed virucidal activity and inhibited the virus at intracellular stages of the viral cycle [76]. Mono and dimethyltryptamine are the main bioactive compounds found in the methanolic extract of *P. viridis* leaves.

The aqueous extract of cranberry pomace showed anti-ZIKV activity (CC_50_ = 865.1 µg/mL, EC_50_ = 26.0 µg/mL, SI= 33.2) in A549 cells. At 100 µg/mL, the extract reduced virus progeny production by 2-log. Moreover, the extract prevented ZIKV entry into the host cell by inhibiting the viral binding step. The extract also showed antiviral effect against different serotypes of DENV [77].

The essential oil of *Lippia alba* showed virucidal activity against ZIKV assayed in Vero cells (CC_50_ = 1789.90 ± 1.74 μg/mL, EC_50_ = 32.20 ± 0.54 μg/mL, SI = 55.6). β-caryophyllene is the major constituent of the essential oil, but it has not been tested, and the virucidal activity of the essential oil was ascribed to the mixture of constituents [60].

The ethanolic extract of *Punica granatum* leaves protected Vero cells against the African MR766 strain of ZIKV (CC_50_ = 123.60 µg/mL, EC_50_ = 11.40 µg/mL, SI = 10.84) [78].

The anti-ZIKV activity of a hydroethanolic extract of *Phyllanthus phillyreifolius* leaves was reported in 4549 cells (CC_50_ = 715.00 μg/mL, EC_50_ = 55.00 μg/mL, SI = 13.00). When tested at 250 μg/mL this extract reduced by 3-log the ZIKV progeny [79].

Some plant extracts have been also reported to be active in vitro against ZIKV, but their selectivity indexes were not determined. Hence, *Carica papaya* fruit pulp inhibited ZIKV infection in A549 cells without loss of cell viability [80]. The extract promoted a concentration-dependent inhibition of ZIKV African strain MR766 (EC_50_ = 300 µg/mL) and reduced the production of ZIKV progeny by 3-log for the Asian strain (PF13) when tested at 1000 µg/mL. In relation to the mechanism of action, papaya pulp extract inhibited the adsorption of ZIKV to A549 cells. Papaya pulp polyphenol-rich extracts fermented with *Lactobacillus plantarum*, *Leuconostoc pseudomesenteroïdes* and *Weissela cibaria* were also tested, producing EC_50_ of 1.5, 1.9 and 4.2 mg/mL, respectively. It was concluded that the lactic fermentation of papaya pulp affects its antiviral activity against ZIKV depending on the bacterial strain used [80].

The ethanolic extract of *Ocimum basilicum* leaves showed anti-ZIKV activity in a concentration dependent manner with EC_50_ of 1:134 and reduced 97% of the ZIKV infectivity in Vero cells. The antiviral effect was ascribed to the ability of *O. basilicum* extract to inhibit ZIKV adsorption to Vero cells [81].

The ethanolic extract of *Passiflora edulis* seeds reduced ZIKV viral load in HTR-8/SVneo cells infected with ZIKV African MR766 and ZIKV Brazilian PE243 strains after 24 h of treatment (MR766 control group 4.77 ± 1.14 × 10^4^ PFU μL^−1^ reduced to 0.64 ± 0.29 × 10^4^ PFU μL^−1^; PE243 control group 5.95 ± 2.08 × 10^5^ PFU μL^−1^ reduced to 0.16 ± 0.06 × 10^5^ PFU μL^−1^), as determined by RT-qPCR. Cells infected with PE243 strain and treated with the extract showed a reduced immunolocalization of NS1 proteins in comparison to the PE243 group, which was observed by flow cytometry and immunofluorescence assay [82].

The ethanolic extracts of the fruits and barks of *Schinus terebinthifolius*, both at 100 μg/mL, showed anti-ZIKV activity, causing a significant reduction in the viral load. The results were obtained by RT-PCRq and flow cytometry in the HTR-8/SVneo cell line. Both extracts were active against two ZIKV strains: African MR766 (control group 69.81 ± 4.19% infected cells; STWF (whole fruits): 47.53 ± 2.14% infected cells; STP (fruits’ peel): 41.90 ± 6.31% infected cells) and Brazilian PE243 (control group: 78.89 ± 2.03% infected cells; STWF (whole fruits): 44.65 ± 7.90% infected cells; STP (fruits’ peel): 51.65 ± 5.84% infected cells). Both extracts did not alter the cell viability of Vero E6, HTR-8/SVneo cells and placental tissue explant culture, tested at concentration from 0.01 to 100 μg/mL [83].

Barbosa et al. (2022) [84] assayed 1000 ethanolic extracts of plants against DENV and ZIKV. Among them, 21 extracts (2.1% of total) presented activity against ZIKV, with EC_50_ values ranging from 10 to 100 µg/mL. In another screening, 37 plant extracts were tested in vitro against Vero cells infected with ZIKV, and significant anti-ZIKV activity was elicited by the extracts of *Maytenus ilicifolia* (4.5 log of inhibition), *Maytenus rigida* (1.7 log of inhibition), *Terminalia phaeocarpa* (3.7 log of inhibition), and *Echinodorus grandiflorus* (1.7 log of inhibition) [85].

The methanolic extracts of aerial parts from *Stenocline ericoides* and *Stenocline inuloides* showed anti-ZIKV activity in A549 cells. They lowered ZIKV progeny production by 2-log when assayed at non-cytotoxic concentrations below 200 µg/mL. *S. ericoides* has virucidal action, whereas *S. inuloides* inhibits the early steps of virus infection. Both extracts also inhibited DENV-2 infection. These extracts are rich in polyphenols and flavonoids and did not exert acute toxicity in Zebrafish [86].

The aqueous extracts of *Artemisia capilaris* and *Hedyotis diffusa* presented antivirus activity against JEV (*Japanese encephalitis virus*), ZIKV, and DENV. They inhibited virus replication and reduced viral RNA levels in a concentration-dependent manner in the range from 0.1 to 10 mg/mL in Vero cells. Both extracts presented anti-*flavivirus* activity in other human cell lines, including human glioblastoma (T98G), human chronic myeloid leukemia (K562), and human embryonic kidney (HEK-293T) cells [87].

Extracts of fresh tomato pomace prepared with ethanol, ethanol/ethyl acetate (1:1), and supercritical fluid extraction with CO_2_ inhibited ZIKV infection up to 75% in a concentration-dependent manner, at 200 μg/mL, in A549 cells. These extracts act on the early stages of the viral cycle by blocking viral entry into the host cell [88].

#### 3.2.2. NPs and Derivatives from Plants

NPs of different classes have been reported to inhibit ZIKV replication in different cell linages, being flavonoids, terpenes, and sesquiterpenes, among others, identified as the most promising compounds.

The phenolic compound nordihydroguaiaretic acid (EC_50_ = 9.1 µM, CC_50_ = 162.1 µM, SI = 17.8), isolated from *Larrea tridentata*, and its semisynthetic derivative tetra-*O*-methyl nordihydroguaiaretic (EC_50_ = 5.7 µM, CC_50_ = 1071.0 µM, SI = 187.9) were reported to inhibit ZIKV-infected Vero cells. Both compounds were also active against *West Nile virus* [89].

Mefloquine, a synthetic derivative of quinine, was found to inhibit ZIKV infection in HeLa cells and primary human amniotic/amnion epithelial cells (HAECs), respectively, at 10 μM and 16 μM [90].

Chloroquine, another derivative of quinine, inhibited ZIKV infection in Vero cells and Huh7 cells, blocking virus internalization (EC_50_ = 4.15 and 2.72 μM, respectively) [91]. Chloroquine did not affect cell viability at concentrations ≤ 50 μM. Chloroquine treatment of Vero cells infected with a Brazilian strain of ZIKV at concentrations ranging from 12.5 to 50 μM increased cell viability from 55% to 100% (CC_50_ = 134.54 ± 16.76 μM, EC_50_ = 9.82 ± 2.79 μM, SI = 13.70). Furthermore, treatment with 25 μM of chloroquine led to a 16-fold reduction in the level of viral RNA detected in the supernatant by RT-qPCR. Chloroquine interferes in the early stages of the ZIKV replication cycle. Other experiments were performed with an African strain in human brain microvascular endothelial cells hBMECs: chloroquine reduced by 45% and 50% the number of ZIKV-infected cells at 25 and 50 μM, respectively, and protected approximately 80% of these cells against infection. In neural stem cells, treatment with 50 μM chloroquine decreased by 57% the number of ZIKV-infected cells and protected 70% of those cells during infection without cytotoxicity effects. In mouse neurospheres, ZIKV infection decreased after treatment with 12.5 μM of chloroquine, according to phase contrast microscopy analysis [92]. Furthermore, hydroxychloroquine tested in vitro by an enzymatic assay inhibited the activity of ZIKV NS2B-NS3 protease [29]. In addition, hydroxychloroquine at 80 μM significantly decreased ZIKV infection in JEG3 cells, reducing both the number and size of foci, according to immunofluorescence microscopy analysis [29].

The flavonoid isoquercitrin showed anti-ZIKV activity in cell lineages A549 (CC_50_ = 551.2 ± 43.2, EC_50_ = 15.5 ± 2.3, SI = 35.6), Huh-7 (CC_50_ = 326.8 ± 45.7, EC_50_ = 14.0 ± 3.8, SI = 23.3) and SH-SY5Y (CC_50_ = 582.2 ± 41.4, EC_50_ = 9.7 ± 1.2, SI = 60.0). Isoquercitrin acts on ZIKV entry in A549 cells, preventing the internalization of viral particles in the host cell [93]. The flavanone naringenin, widely found in citrus fruits, tomatoes, cherries, grapefruits, and cocoa, showed activity against distinct ZIKV strains (Brazilian, Asian and African strains) in a concentration-dependent manner in A549 human cells. Narigenin was shown to be active against the Brazilian strain (CC_50_ = 693.60 μM, EC_50_ = 58.79 μM and SI = 11.79 as well as against the Asian (~4 fold reduction in infection) and African lineages (~2 fold reduction in infection), which were both tested at 125 μM. The antiviral activity of naringenin was also observed in primary human monocyte-derived dendritic cells infected with ZIKV. The mechanism of action indicated that naringenin acts on viral replication or viral particle assembly [37].

Two other flavones, baicalein (EC_50_ = 0.004 µM, CC_50_ = 420.000 µM, SI = 105) and baicalin (EC_50_ = 14.0 µM, CC_50_ = 553.0 µM, SI = 40), decrease ZIKV replication significantly. Baicalein was more effective against post-entry replication of ZIKV, while baicalin acted majorly at the stage of virus entry into the host cell [46]. Some semisynthetic derivatives of baicalein were also described to possess antiviral activity. Hence, a derivative obtained by the introduction of a thiocarbonyl group at the C-4 position and another prepared by setting a halophenylamino group at C-4′ showed potent antiviral activities against CHIKV, WNV (*West Nile virus*) and ZIKV, and were shown to be 5–10 times more potent than baicalein. Both derivatives showed CC_50_ of 100.0 μM and EC_50_ = 3.8 μM and EC_50_ = 1.9 μM, respectively, in Huh7 cells [94].

The dihydrochalcone phloretin, found in apple and pear trees, decreased the infectious titers of ZIKV African strain MR766 (EC_50_ = 22.85 ± 5.20 M, SI = 4.40 ± 0.80) and PRVABC59 ZIKV clinical isolate from Puerto Rico (EC_50_ = 9.31 ± 2.50 M, SI = 10.70 ± 2.20). Phloretin acts in several stages of ZIKV replication, including RNA production, and in a later stage of propagation, such as assembly or exit. Phloretin decreased the apoptotic activity of caspase-3 and caspase-7 and reduced the phosphorylation of Akt/mTOR pathways. In addition, phloretin acts inhibiting the glucose uptake, thus reducing the spread of ZIKV [95].

The isoquinoline alkaloid berberine, isolated from *Berberis vulgaris*, exhibited virucidal effect in vitro (CC_50_ = 221.00 μM, EC_50_ = 39.06 μM, SI = 5.65) by interfering directly with ZIKV viral particles, as similarly observed for emodin (CC_50_ = 68.60 μM, EC_50_ = 3.20 μM, SI = 21.44), which is an anthraquinone found in the Chinese herbs *Rheum palmatum*, *Polygonum multiflorum*, *Aloe vera* and *Cassia obtusifolia* [96]. The virucidal effect of berberine has been also reported against ZIKV cultured in BHK-21 cells (EC_50_ = 11.42 µM, CC_50_ = 115.00 µM) [97].

Silymarin, a flavonolignan extracted from seeds of *Silybum marianum*, showed antiviral effect against ZIKV (CC_50_ = 596.19 μg/mL, EC_50_ = 34.17 μg/mL, SI = 17.45) in Vero cells. When assayed at 100 μg/mL, it reduced the viral load by 2-log. Silymarin acts in the stages of viral adsorption and internalization and showed virucidal effect on Zika viral particles [98].

The alkaloids lycorine and cherylline, isolated from *Crinum jagus*, were active against ZIKV in Huh 7.5 cells (SI = 35.4 and SI >12.3, respectively) [99], along with warifteine, isolated from *Cissampelos sympodialis*, tested in ZIKV-infected Vero cells (SI = 68.3) [100].

Ellagic acid, isolated from *Punica granatum*, was active against the Asian strain HPF2013 of ZIKV (CC_50_ = 446.85 µM, EC_50_ = 30.86 µM, SI = 14.50) and MR766 strain (CC_50_ = 446.85 µM, EC_50_ = 46.23 µM, SI = 9.70). When tested at 109.2 and 36.4 µM, it reduced ZIKV progeny by 1–2-log. The CC_50_ values of the ethanolic extract of *P. granatum* and ellagic acid were ≥100 µg/mL [78].

The chemical composition of the hydroethanolic extract of *Phyllanthus phillyreifolius* comprises geraniin, the major constituent, along with ellagic acid, elaeocarpusin, rutin, quercetin, and gallic acid. Geraniin showed anti-ZIKV activity in A549 cells (CC_50_ = 420 μg/mL, EC_50_ = 22 μg/mL, SI = 19). The extract of *P. phillyreifolius* and geraniin exerted antiviral effect after the virus bound to the cell membrane, and it was explained by impairing the ZIKV-bound particles to be internalized into the host cell [79].

Polyphenols—especially flavonoids—are plant secondary metabolites potentially active against ZIKV, as highlighted by in silico studies. The flavonoids galangin (EC_50_ = 14.36 ± 9.50 μM, CC_50_ = 227.20 ± 84.30 μM, SI = 16), kaempferide (EC_50_ = 5.83 ± 1.92 μM, CC_50_ > 500 μM, SI > 86), quercetin (EC_50_ = 2.30 ± 0.50 μM, CC_50_ > 500 μM, SI > 218), myricetin (EC_50_ = 0.58 ± 0.17 μM, CC_50_ > 500 μM, SI > 862), and epigallocatechin gallate (EC_50_ = 0.020 ± 0.003 μM, CC_50_ > 500 μM, SI > 25,000) showed anti ZIKV effects in a concentration-dependent manner in Vero cells. The time-of-addition assay indicates that these flavonoids exerted their antiviral activities during the early stages of ZIKV infection [101].

Diterpenes of the abietane type, commonly found in conifers, angiosperm species, and in plants of the families Araucariaceae, Cupressaceae, Pinaceae, and Podocarpaceae were evaluated for their antiviral activity against ZIKV in Vero cells. Semisynthetic derivatives of abietanes were prepared from (+)-dehydroabietylamine, producing the analogues 18-aminoferruginol, hydroxy-N, *N*-phthaloyldehydroabietylamine, 12-acetoxy-N, *N*-(tetrachlorophthaloyl)-dehydroabietylamine, 12-hydroxy-N-tosyldehydroabietylamine, 18-oxoferruginol, and 12-nitro-N-benzoyldehydroabietylamine. Among them, 18-oxoferruginol showed the most promising anti-ZIKV activity in Vero cells (CC_50_ = 35.09 ± 5.20, EC_50_ = 2.60 ± 0.07, SI = 13.51) [102].

The cardiotonic steroid ouabain induced a concentration-dependent inhibition of ZIKV when assayed both at pre-treatment (CC_50_ = 68.90 nM, EC_50_ = 2.33 nM, SI = 29.50) and post-treatment (EC_50_ = 1.92 nM, SI = 35.80) conditions. The reduction in virus infectious was accompanied by a decrease in ZIKV RNA levels on post-treatment condition at all tested concentrations, with percentage reductions of 65.6%, 71.9%, 71.3% and 78.1%, respectively, at 2.5, 5, 10 and 20 nM. The authors suggested that the mechanism of ZIKV inhibition by ouabain occurred at the replication step [103].

Two flavaglines isolated from *Aglaia* spp. inhibited ZIKV replication in A549 cells, namely silvestrol (CC_50_ = 9.42 nM, EC_50_ = 1.08 nM, SI = 8.72) and rocaglate CR-31-B (-) (CC_50_ =19.34 nM, EC_50_ = 1.13 nM, SI = 17.11) [104]. Eucalyprobusone G, a symmetrical acylphloroglucinol dimer from *Eucalyptus robusta*, induced significant ZIKV inhibition in Vero cells, without cytotoxicity, with SI = 581 [105]. The chalcone 4-hydroxyderricin, isolated from *Angelica keiskei*, inhibited ZIKV replication in Vero cells (EC_50_ = 6.6 μM, CC_50_ = 103.0, SI = 15.6) [58]. Diphyllin is an arylnaphtalide lignan extracted from plants used in the traditional Chinese medicine and exerted antiviral activity against ZIKV (Paraiba 01 strain) in Vero cells (EC_50_ = 0.54 μM, CC_50_ > 100 μM and SI > 185.19). When tested at concentrations between 12.5 and 25 μM, the viral surface antigen expression was eliminated in Vero cells [106].

The antiviral activity of cinnamic acid was evaluated in different cell lines, including Vero (CC_50_ > 4000 μM, EC_50_ = 49.55 μM, and SI > 80), Huh-7 (CC_50_ = 3752.00 μM, EC_50_= 83.96 μM, and SI = 44.69) and A549 (CC_50_ = 1665 μM, EC_50_= 103.8 μM, and SI = 16.04) cells. The time-of-addition assay indicated that cinnamic acid probably exerts antiviral activity in the post-entry stage. An inhibition assay of ZIKV NS5 RdRp was performed and elicited 50% of inhibition when cinnamic acid was tested at 255.7 μM [107].

Glycyrrhetinic acid is a triterpene isolated from *Glycyrrhiza glabra* and *Glycyrrhiza uralensis*. This compound and some semisynthetic derivatives were tested in vitro against SF268 cells infected with ZIKV. The best results were obtained for the glycyrrhetinic acid derivatives 3-*O*-acetyl-30-aminopyridine (CC_50_ > 50 µM, EC_50_ = 0.13 ± 0.01 µM, SI > 384), 3-semicarbazone-30-butyl (CC_50_ > 50 µM, EC_50_ = 0.55 ± 0.74 µM, SI > 90.91), 1,2,3-thiadiazole-30-methyl (CC_50_ > 50 µM, EC_50_ = 0.29 ± 0.05 µM, SI > 172.41) and 1,2,3-thiadiazole-30-butyl (CC_50_ > 50 µM, EC_50_ = 0.56 ± 0.42 µM, SI > 89.28). The time-of-addition assay suggests that the four glycyrrhetinic acid derivatives inhibited the entry and post-entry stages of ZIKV infection [44].

A set of glycyrrhizic acid derivatives conjugated with amino acids was tested against ZIKV-infected SF268 cells and other cell lines. The derivatives bearing two amino acid methyl ester residues in the sugar unit, along with those possessing two amino acid ethyl ester residues in the carbohydrate chain, showed values of EC_50_ between 0.09 ± 0.08 μM and 2.23 ± 0.72 μM and induced a concentration-dependent reduction in the cytopathic effect in infected cells. Of note, glycyrrhizic acid conjugated with amino acids and their esters aspartate (methyl ester)-methyl ester (EC_50_ = 0.23 ± 0.04 μM) and tyrosine methyl ester (EC_50_ = 0.09 ± 0.02 μM) showed the best inhibitory activity against ZIKV infection. A time-of-addition/removal assay carried out with both derivatives indicated that they may act inhibiting the entry stage and blocking the post-entry stage, respectively. Moreover, docking studies revealed that both compounds interact with the active pocket of NS5 MTase [108].

The anti-ZIKV activity of lycorine was assessed in different cell lines such as Vero (CC_50_ = 21.00 μM, EC_50_= 0.39 μM, and SI = 53.84), A549 (CC_50_ = 4.29 μM, EC_50_= 0.22 μM, and SI = 19.50), and Huh7 cells (CC_50_ = 4.40 μM, EC_50_= 0.22 μM, and SI = 20.00). The time-of-addition/time-of-removal assay pointed out that lycorine probably exerts antiviral activity in post-entry events of the ZIKV replication cycle [50].

As described in the previous paragraphs, the antivirus effect of NPs and derivatives has been assayed majorly on different cell lineages infected with ZIKV. Only two articles were identified reporting the effect of NPs on isolated ZIKV proteins. The following compounds, isolated from *Codiaeum peltatum* extract, exhibited significant inhibitory activity against ZIKV-NS5 RNA-dependent RNA polymerase: actephilol B3 (IC_50_ = 8.1 ± 0.9 μM), actephilol C (IC_50_ = 10.9 ± 2.5 μM), actephilol A (IC_50_ = 4.8 ± 0.9 μM), epi-actephilol A (IC_50_ = 8.1 ± 1.0 μM), fimbricalyx C (IC_50_ = 13.6 ± 1.2 μM), and fimbricalyx D (IC_50_ = 12.7 ± 1.3 μM), evaluated by enzymatic assays [109]. These compounds were also active against DENV-2 [109]. Another publication addressed the effect of different chalcones on ZIKV NS5 RdRp activity and disclosed only xanthoangelol as a potential allosteric inhibitor (IC_50_ = 6.9 µM), which was evaluated by ZIKV enzymatic protease assays [58].

Some compounds herein described and/or those enclosed in Table 1 do not act solely at one stage of the ZIKV replication cycle but may target different stages. For example, (-)-epigallocatechin gallate elicits virucidal effect by targeting both viral entry and NS2B-NS3 protease. This bifunctional feature could be advantageous for drug development, since dual effects are achieved for one single compound [110]. It should be remembered that NPs offer a vast and unexplored diversity of chemical structures unmatched by even the biggest combinatorial databases [111]. Jointly, these features reinforce the relevance of searching NPs to identify new bioactive antiviral compounds that are potentially useful for drug development.

Table 1 summarizes some selected NPs and derivatives that showed in vitro activity against ZIKV for which studies to investigate the mechanism of action were performed, and the antiviral activity was evaluated using different assays.

**Table 1:** In vitro anti-ZIKV activity of selected NPs and derivatives evaluated by different assays.

Classes of NP	Compound	Cell Strain/Methods/Observed Activity/Additional Comments/(Reference)
Flavonoids	Apigenin	Vero CCL-81 cells. Lysis plate reduction assay. Inhibition of ZIKV infection (>100 µM) [41]
	Bereberine derivative	Vero cells. Immunofluorescence assay. African ZIKV strain MR766. CC_50_ = 80.9 ± 5.7 μM, EC_50_= 5.3 ± 1.9 μM, SI = 15.3 [32]
	Curcumin	Vero cells. Lysis plate reduction assay. Concentration-dependent antiviral activity from 12.50 to 50.00 μM. It promoted antiviral mainly on the viral attachment [36]
	Curcumin, bisdemethoxycurcumin, demethoxycurcumin, fractions EF-24 and FLLL31	HeLa cells. Lysis plate reduction assay. EC_50_ (μM) = 1.90; 3.61; 5.91; 1.49 and 6.85, CC_50_ (μM) = 11.60; 16.00; 13.20; 3.46 and 3.53 Viral replication reduction by inhibiting viral binding at the cell surface [112]
	Derivative of houttuynoids (flavonoid glycoside)/tetra-acetyl-houttuynoid B	A549 cells. Lysis plate reduction assay. Polynesia strain: EC_50_ = 1.675 μM Uganda strain: EC_50_ = 1.552 μM and CC_50_ = 6.3 μM. Blockade the internalization of ZIKV [113]
	(−)-Epigallocatechin gallate	Vero E6 cells. Lysis plate reduction assay. African strain: virucidal effect at 200 µM. Inhibition of ZIKV adsorption at 25 µM. Brazilian strain: inhibition of ZIKV adsorption at 50 µM [114]
	(−)-Epigallocatechin gallate (EGCG) and delphinidin (D)	Vero cells. Lysis plate reduction assay. 10 μM of EGCG or D. Virucidal effect [115]
	Galagin, kaempferide; quercetin; myricetin, and epigallocatechin gallate (EGCG)	ZIKV NS2B-NS3 protease inhibition assay. IC_50_ (μM) = 25.68 ± 9.17; 7.18 ± 2.16; 1.17 ± 0.22; 1.10 ± 0.88; and 0.73 ± 0.22 [101]
	Gossypol, curcumin, digitonin, and conessine	Vero E6 cells. Lysis plate reduction assay and ELISA CC_50_ = 14.17 to 323.71 µM Panama strain (2015). EC_50_ = 3.95 to 13.71 μM Panama strain (2016). EC_50_ = 3.48 to 13.67 μM Mexico strain. EC_50_ = 4.20 to 14.04 μM Colombia strain. EC_50_ = 0.21 to 16.57 μM Honduras strain. EC_50_ = 2.28 to 11.60 μM Puerto Rico strain. EC_50_ = 3.76 to 12.85 µM Thailand strain. EC_50_ = 1.98 to 10.84 µM Nigeria strain. EC_50_ = 3.31 to 55.30 µM Mosquito strain. EC_50_ = 2.79 to 10.94 µM African strain. EC_50_ = 3.75 to 11.42 µM [116] Gossypol induced virucidal effect bonding to ZIKV protein E. Curcumin acts on viral adsorption stage. Digitonin and conessine act on viral adsorption and internalization [116]
	Hesperetin	Vero E6 cells. ZIKV NS2B-NS3 protease inhibition assay. EC_50_ = 12.6 ± 1.3 µM. Inhibition the NS2B/NS3 protease [38]
	4-hydroxyderricin, xanthoangelol and xanthoangelol-E	ZIKV NS2B-NS3 protease inhibition assay. IC_50_ (μM) = 47.00 ± 10.00; 50.00 ± 5.00 and 18.00 ± 5.00. Antiviral effect at 200 μM [58]
	Irigenol hexa-acetate, katacine, epigallocatechin gallate, epicatechin gallate, theaflavin gallate, pyrogallin, hematein, tannic acid, sennoside, gossypol, ursololactone and juglone	ZIKV NS2B-NS3 protease inhibition assay. IC_50_ (μM) = 0.26; 0.30; 0.14; 0.46; 0.43; 2.30; 0.34; 0.02; 0.66; 0.60; 1.00 and 5.70 [117]
	Luteolin, myricetin, astragalin, rutin, epigallocatechin gallate, epicatechin gallate, gallocatechin gallate	ZIKV NS2B-NS3 protease inhibition assay IC_50_ (μM) = 53.0 ± 1.3; 22.0 ± 0.2; 112.0 ± 5.5; 104.0 ± 2.9; 87.0 ± 1.2; 89.0 ± 1.6 and 99.0 ± 1.8 [118]
	Pinocembrin	JEG-3 cells. Lysis plate reduction assay, qRT-PCR and Western blot. EC_50_ = 17.44 µM, CC_50_ = 250.70 µM. Inhibition ZIKV replication cycle, inhibits viral RNA production and envelope protein synthesis [119]
	Quercetin	ZIKV NS2B-NS3 protease inhibition assay. IC_50_ = 26.0 ± 0.1 µM; K_i_ = 23.0 ± 1.3 µM [120]
	Quercetin-3-β-*O*-D-glucoside	Vero cells. qRT-PCR. Canadian strain: EC_50_ = 1.2 μmol/L [121]
	Resveratrol (RES)	Vero cells. Foci forming assay and qRT-PCR. At 20, 50, 80 and 100 µM, RES reduced the foci forming by 25%, 76%, 93% (1 log) and 97% (1 log). Virucidal activity by possible interference on ZIKV binding to host cells [122]
	Resveratrol	ARPE-19 and hTERT-RPE-1 cells. Lysis plate reduction assay and immunofluorescence assay. RES (50 μM) attenuates the cytopathic effect in both cells. Plaque assay. RES (50 μM) decreases 70% production of viral particles. RES prevents ZIKV-induced mitochondrial disruptions [64]
	Rutin, quercetin, and pedalitin isolated from *Pterogyne nitens*	ZIKV NS2B-NS3 protease inhibition assay. IC_50_ (μM) = 139.0 ± 26.0; 34.0 ± 5.0, and 5.0 ± 1.0, respectively Vero cells. ZIKV activity of quercetin (250 µM) and pedalitin (500 µM) near cytotoxic concentration (>250 µM) [39]
	Pedalitin and quercetin	ZIKV NS5 RdRp inhibition assay. IC_50_ = 4.1 (pedalitin) and 0.5 µM (quercetin) [123]
	Sophoraflavenone G isolated from *Sophora flavecens* roots	A549 cells. Flow cytometry. EC_50_ = 22.61 µM, CC_50_ = 58.21 µM Viral RNA polymerase inhibition [124]
	Theaflavin	ZIKV NS5 Methyltransferase inhibition assay. IC_50_ = 10.10 μM Anti-ZIKV activity assay. Huh-7 cells. EC_50_ = 8.19 μM and CC_50_ > 128 μM [52]
	Theaflavin-3,3′-digallate	Vero E6 cells. qRT-PCR and Western blot. EC_50_ = 7.65 μM. Inhibition of ZIKV polyprotein precursor cleavage by NS2B-3 protease, blocking virus growth [125]
	Theaflavin-3,3′-digallate, mebromin, tannic acid, gossypol acetic acid, grape seed extract, 1,2,3,4,6-*O*-pentagalloylglucose and 6-tridecylsalicylic acid	ZIKV NS2B-NS3 protease inhibition assay. IC_50_ (μM) = 2.30, 9.17, 2.27, 9.79, 12.79, 3.61, and 1.42 [125]
	Xanthoangelol	ZIKV NS5 RdRp inhibition assay. IC_50_ = 6.9 ± 0.9 μM Antiviral effect at 20 µM [58]
Steroids	7-Dehydrocholesterol	A549 cells. PFU by qRT-PCR. Enhancing of IFN-I production reducing copies of ZIKV [126]
	Dehydroepiandrosterone and derivatives (AV1003, AV1004 amd AV1017)	Vero cells. PFU by qRT-PCR. Antiviral activity. EC_50_ (μM) = 0.34, 5.56, and 6.31. Time of addition and Western blot. Inhibitory effect after viral infection [127]
	Derivatives of dehydroepiandrosterone (HAAS-AV3026 and HAAS-AV3027)	Vero cells. Lysis plate reduction assay, immunofluorescence assay, qRT-PCR and Western blot. SI = 19.23 for HAAS-AV3026 and SI = 20.62 for HAAS-AV3027. Inhibition of viral infection, protein production, and viral RNA synthesis [128]
	25-Hydroxycholesterol	Vero cells. Lysis plate reduction assay and immunofluorescence assay. EC_50_ = 188 nM HCO cells. Inhibition of ZIKV viral entry. Reduction (90%) of ZIKV genomic RNA [129]
	Silvestrol	A549 cells. Inhibition of ZIKV replication at 5 nM and 50 nM PHHs cells. Blockade of the ZIKV genome translation at 5 nM, 10 nM and 100 nM [130]
Alkaloids	Cephalotaxine isolated from *Cephalotaxus drupacea*	Vero cells. Lysis plate reduction assay, qRT-PCR and Western blot. SI CC_50_ > 300 µM. Suppression (98%) of ZIKV RNA production at 100 μM Reduction in ZIKV actin protein expression. Inhibition of ZIKV production and replication. Virucidal activity [131]
	Chloroquine	Vero cells. qRT-PCR. Chinese strain: EC_50_ = 4.15 μM. Huh7 cells: EC_50_ = 1.72 μM. Cambodian strain: EC_50_ = 2.72 μM. Suppression of ZIKV internalization [90]
	Harringtonine	Vero cells. RT-qPCR, Western blot and fluorescent focus assay Interference in the binding, entry, replication, and release stage of ZIKV life cycle. Virucidal effect [31]
	Homoharringtonine (HHT), bruceine D (BD), dihydroartemisinin (DHA), and digitonin (DGT)	BHK-DR cells. Lysis plate reduction assay, immunofluorescence assay and Western blot. EC_50_ (μmol/L) = 0.19, 0.36, 0.47, and 0.62. CC_50_ (μmol/L) = 50.66, 25.66, >100, and >100 HHT, BD and DHA inhibited ZIKV infection at the post-entry stage and DGT acts on early stage of the ZIKV infection [132]
	Lycorine	Vero cells. PFU by qRT-PCR, immunofluorescence assay and Western blot. RNA synthesis and virion formation inhibition. Total plaque formation inhibition at 5 μM. Significant reduction in ZIKV NS5 protein and ZIKV envelope [50]
	Palmatine	Vero cells. Lysis plate reduction assay, immunofluorescence assay and qRT-PCR. Reduction in viral RNA and viral progeny by 90% at 80 mM. It affected virus binding, internalization, and stability through binding to ZIKV envelope proteins [30]
Lipopeptide	Anidulafungin	Vero cells. Immunofluorescence assay. ZIKV infection inhibition in a concentration-dependent manner. RT-qPCR, Western blot, and TCID_50_ assay. Significant decrease in RNA expression and in ZIKV progeny yield [133]
Phenolic acid	Cinnamic acid	Vero cells. Immunofluorescence assay, Western blot and PFU assay. Inhibition of ZIKV infection. Concentration-dependent reduction in E and NS5 ZIKV proteins. Inhibition of plaque formation at 100 μM [107]
	Rottlerin	A549 cells. Focus forming assay, qRT-PCR and Western blot. CC_50_ = 47.78 μmol/L, EC_50_ = 1.06 μmol/L. Huh7.5 cells, EC_50_ = 0.70 μmol/L SNB19 cells. EC_50_ = 0.19 μmol/L Pre-treatment disturbs the endocytosis of ZIKV. Post-treatment alter the stage ZIKV maturation [134]
	Ginkgolic acid	Vero cells. Lysis plate reduction assay and immunofluorescence assay. Virucidal effect at 10 µM [135]
Terpenes	3α-Cinnamoyl-28-nor-D-friedoolean-14-en-16-one-23-oic acid and 2-deoxyphorbol-13-(*Z*)-5-tetradecanoate isolated from *Stillingia loranthacea* root bark	Vero cells. Foci forming assay. (CC_50_ = 50.2 ± 1 µM and 60.5 ± 2 µM). Reduction in ZIKV replication (1.7 and 1.8 log TCID_50_/mL) [136]
	Glycyrrhetinic acid derivatives	SF268 cells. Lysis plate reduction assay and immunofluorescence assay. Cytopathic effect. All samples inhibit ZIKV-induced cytotocicity Inhibition of the ZIKV NS1 protein expression in both assays in a concentration-dependent manner [44]
	3-(*E*)-2-(1*R*,4a*S*,5*S*,8a*R*,*E*)-6-(hydroxyimino)-5,8a-dimethyl-2-methylene-5-(trityloxy)-methyl)decahydronaphthalen-1-yl)vinyl)furan-2(5H)-one, a derivative from andrographolide (*Andrographis paniculata*)	Vero cells, Huh7 cells, and A549 cells. qRT-PCR, immunofluorescence assay and Western blot. CC_50_ > 200 μM for all cell lines. Inhibition of ZIKV infection (EC_50_ = 0.48, 0.37 and 0.45 μM). ZIKV E protein totally inhibited at 0.40 μM. Inhibition of post-entry stage of ZIKV life cycle. Inhibition of ZIKV NS5 methyltranferase [137]
	Neoandrographolide	Vero CCL-81 cells. Lysis plate reduction assay. Inhibition of ZIKV infection (>100 µM) [41]
Saponin	Eleutheroside B	Vero CCL-81 cells. Lysis plate reduction assay. Inhibition of ZIKV infection (>100 µM) [41]

CC_50_ = median cytotoxic concentration; EC_50_ = median effective concentration; IC_50_ = median inhibitory concentration; Ki = inhibition constant. Vero = kidney epithelial cells of African green monkey; A549 = human epithelial cells; BHK-DR = generated from BHK-21 (baby hamster kidney cell) cells as parental cells; HCO = human cortical organoid cells; HeLa = human epithelial cell line derived from a cancerous tumor of the cervix; Huh-7 = human epithelial-like, hepatoma cells; JEG-3 = human choriocarcinoma placental cells; ARPE-19 and hTERT-RPE-1: cell lines from retinal pigment epithelium; PHHs = primary human hepatocytes cells; SF268 = glioma cells; SNB19 = glioblastoma cells. PFU = plaque-forming unit; TCID = tissue culture infectious dose. Some articles do not describe CC_50_, EC_50_ and SI data.

#### 3.2.3. NPs from Bacteria

Although less numerous than NPs from plants, several compounds obtained from different bacterial strains have been reported to inhibit ZIKV replication. Duramycin is a cyclic peptide produced by *Streptomyces cinnamoneus*, which is capable of binding to phosphatidylethanolamine found in enveloped virions, thus preventing TIM1 binding. It reduced ZIKV infection by >50% in primary human placental cells and by >95% in primary cells from chorionic villi when assayed respectively at 0.1 and 0.2 µM. Furthermore, duramycin at 1 µM reduced by 2-log the viral load in amniochorionic membranes [138,139].

Nanchangmicin, a polyether produced by *Streptocyces nachangensis*, was characterized as an inhibitor of ZIKV entry (via clathrin-mediated endocytosis) into different cell lines, including Vero, U2OS (human osteosarcoma, EC_50_ = 0.1 μM), HBMEC (human microvascular endothelial cells, EC_50_ = 0.4 μM), JEG-3 (primary human trophoblast cells, EC_50_ = 0.97 μM), UtMEC (human uterine microvascular endothelial cells), primary placental fibroblasts, and HUVEC (human umbilical vein endothelial cells). The antiviral effect on the last two cell lineages was observed by confocal microscopy images, and the EC_50_ were not reported [140]. Interestingly, nanchangmicin was also active against *West Nile*, *Dengue* and *Chikungunya* viruses, which possess a similar cell entry route of ZIKV [140].

Novobiocin is a coumarin-derived antibiotic obtained from *Streptomyces niveus*. It was shown to be active against ZIKV in Vero cells (CC_50_ = 850.50 μg/mL, EC_50_ = 26.12 ± 0.33 μg/mL, SI = 32.56) and in Huh-7 cells (CC_50_ = 1103.18 μg/mL, EC_50_ =38.14 ± 4.53 μg/mL, SI = 28.92). In the plaque reduction assay, novobiocin promoted 100% plaque reduction at concentrations ≥50 μg/mL (EC_50_ = 15.21 ± 0.14 μg/mL). It inhibited the ZIKV NS2B-NS3 protease in vitro (IC_50_ = 14.2 ± 1.1 µg/mL) and interfered with the stages after ZIKV internalization [43].

SH-SY5Y and A549 cells pre-treated with bafilomycin A1 (10 nM), a macrolide antibiotic produced by different *Streptomycetes* species, reduced viral RNA of African and Asian ZIKV strains, as determined by RT-qPCR. The mechanism of action investigated in A549 cells showed that bafilomycin A1 inhibits ZIKV adsorption to the host cell, preventing the spread of infection and interfering with viral maturation [141].

Antimycin A produced by *Streptomyces* sp. (CC_50_ = 51.28 µM, EC_50_ = 2.00 µM, SI = 25.64) and pyrazofurin found in *Streptomyces candidus* (CC_50_ = 270.60 µM, EC_50_ = 5.96 µM, SI = 45.35) showed antiviral effect against ZIKV-infected Vero cells under post-treatment conditions. Both compounds induced an anti-ZIKV effect in A549 cells (EC_50_ = 1.71 µM, SI = >29.24 to antimycin A; EC_50_ = 0.33 µM, SI = 151.52 to pyrazofurin). They were also shown to inhibit different ZIKV strains, including African, Asian, and American strains, thus characterizing a broad spectrum of action [142].

Labyrinthopeptins A1 and A2 (LabyA1 and LabyA2) are post-translationally modified peptides isolated from the actinomycete *Actinomadura namibiensis* DSM 6313. LabyA1 was considered a potent inhibitor of African (ZIKV-976) and Asian (ZIK-H/PF/2013) ZIKV strains with EC_50_ values of 2.0 μM and 1.6 μM, respectively, whereas LabyA2 also inhibited both strains but was less potent (EC_50_ values of 9.6 and 3.3 μM, respectively, for the African and Asian strains), which were both evaluated in Huh-7 cells [143]. LabyA1 was also active against DENV-2, West Nile virus, Hepatitis C virus, Herpes simplex virus, Human immunodeficiency virus, Human cytomegalovirus, and Chikungunya virus [143]. Another study demonstrated the anti-ZIKV potential of Laby A1 against different strains of ZIKV in Vero cells with CC_50_= 124.6 ± 3.1 μM: ZIKV IBH 30,656 (EC_50_ = 0.51 ± 0.06 μM, SI = 245), ZIKV MR766 (EC_50_ = 0.99 ± 0.1 μM, SI = 126), ZIKV PRVABC59 (EC_50_ = 0.74 ± 0.12 μM, SI = 169), and ZIKV FLR (EC_50_= 0.80 ± 0.18 μM, SI = 156). The results of the time-of-drug addition assay suggested that Laby A1 interferes in the viral entry step [144].

The alkaloid anisomycin, an antibiotic retrieved from bacteria of the genus *Streptomyces*, such as *S. griseolus* and *S. hygrospinosus*, inhibited ZIKV replication in Vero cells infected with African (EC_50_ = 15.9 ± 1.1 nM, SI = 340 ± 30) and Asian (EC_50_ = 50.7 ± 1.0 nM, SI = 107 ± 9) strains, along with a Brazilian clinical isolate (EC_50_ = 33.0 ± 1.1 nM, SI = 163 ± 9) and showed CC_50_ of 5400 ± 100 nM in Vero cells [145]. The antiviral effect of anisomycin was also observed in human cells A549 (EC_50_= 7.9 ± 1.2, SI > 11,900) and U937 (EC_50_ = 30.9 ± 1.0, SI = 14 ± 2) infected with the Asian strain of ZIKV. The initial stages of the ZIKV replicative cycle were not affected, and a high inhibition of ZIKV viral protein expression was demonstrated after treatment with anisomycin. Anisomycin also presented antiviral activity against DENV [145].

Additionally, ascomycin was able to inhibit ZIKV infection in Vero cells (EC_50_ = 0.11 μM, CC_50_ = 21.32 μM, SI = 193.82) and in human lineages such as glioblastoma SNB-19 cells (EC_50_ = 0.06 μM, CC_50_ = 47.93 μM, SI = 798.83) and hepatoma Huh7 cells (EC_50_ = 0.38 μM, CC_50_ = 49.68 μM, SI = 130.74). Results of the time-of-addition study indicated that ascomycin mainly affects the virus replication step [146].

Compounds isolated from *Streptomyces* spp., berninamycin F, berninamycins G–I, and berninamycin derivatives C and D showed anti-ZIKV activity in Vero cells with EC_50_ values (μmol/L) of 10.45 ± 0.15; 7.38 ± 0.45; 5.52 ± 0.60; 6.08 ± 0.53; 6.93 ± 0.82 and 4.37 ± 0.53, respectively. All six compounds showed CC_50_ > 100 μmol/L [147].

#### 3.2.4. NPs from Fungi

Fungi are recognized sources of bioactive compounds potentially useful to be developed as antivirals. Although promising, the research of fungal compounds with antiviral activity is still an emerging field of investigation [111].

The fermentation products of *Penicillium brevicompactum*, mycophenolate mofetil (CC_50_ = 166.30 µM, EC_50_ = 3.52, µM SI = 47.24) and mycophenolic acid (CC_50_ = 275.40 µM, EC_50_ = 4.26 µM, SI = 64.65) showed antiviral effect against ZIKV-infected Vero cells under post-treatment conditions. The compounds also induced anti-ZIKV effect in A549 cells (CC_50_ ≥ 50 µM, EC_50_ = 0.83 µM, SI ≥ 60.24 to mycophenolate mofetil, CC_50_ ≥ 50 µM, EC_50_ = 0.65 µM, SI > 76.92 to mycophenolic acid). Potent anti-ZIKV activity was also observed for mycophenolate mofetil (CC_50_ = 166.30 µM, EC_50_ = 8.51 µM, SI = 20.51) and mycophenolic acid (CC_50_ = 275.40 µM, EC_50_ = 7.58, SI = 36.33) under pre-treatment conditions. Mycophenolate mofetil also exhibited potent anti-ZIKV activity under co-treatment conditions (CC_50_ = 166.30 µM, EC_50_ = 2.27, SI = 73.26). Interestingly, these compounds were capable of inhibiting different ZIKV strains, including African, Asian, and American strains, thus characterizing a broad spectrum of action [142].

Cavinafungin is a lipopeptide isolated from *Colispora cavincula*. This compound was reported to be a potent inhibitor of ZIKV multiplication in A549 cells (EC_50_ = 150 ± 23 nM, CC_50_ = 1650 ± 49 nM, SI = 11), acting as a selective inhibitor of viral peptidase signaling [148]. It also inhibited in vitro the replication of four DENV serotypes. Brefeldin A is a secondary metabolite isolated from the ethanolic extract of the culture broth of *Penicillium* sp. FKI-7127. When tested against ZIKV in Vero cells, brefeldin A induced a strong inhibition in a concentration-dependent manner (EC_50_ = 54.8 ± 0.4 nM, CC_50_ = 2000.0 nM, SI = 36.5). Brefeldin A was also active against four serotypes of dengue and *Japanese encephalitis virus* [149].

β-1,3-β-1,6-Glucan isolated from *Agaricus subrufescens* fruiting bodies (FR) and its sulfated derivative (FR-S) were evaluated as potential anti-ZIKV agents. FR-S was able to inhibit ZIKV replication in human monocytic cells when added simultaneously with viral infection (CC_50_ > 500 μg/mL, EC_50_ = 188.70 ± 12.87 μg/mL, SI > 2.78), whereas FR was inactive. Additionally, no inhibitory effect was observed when either FR-S or FR were added post-infection. The effect of these compounds on permeability was assayed in vitro in human pulmonary microvascular endothelial cells (HPMECs) to assess their capacity of protecting against endothelial barrier dysfunction induced by ZIKV. FR and FR-S, tested at 0.12 μg/mL, promoted 100% inhibition of ZIKV NS1-induced hyperpermeability, as measured by trans-endothelial electrical resistance [150].

#### 3.2.5. NPs from Marine Organisms

The marine environment offers a unique ecosystem for identifying new compounds as potential therapeutic drugs, although it is currently an under-exploited resource. Compounds obtained from marine organisms may induce antiviral activity with unique mechanisms of action [151].

The dichloromethane extract of the marine seaweed *Canistrocarpus cervicornis* inhibited ZIKV replication in a concentration-dependent manner in Vero cells (EC_50_ = 2.15 ± 0.22 μg/mL, CC_50_ = 438.00 ± 5.18 μg/mL, SI = 203.72). Dolastane, a diterpene isolated from this extract, induced higher activity against ZIKV than the extract (EC_50_= 0.75 ± 0.18 µM, CC_50_ = 935.00 ± 11.00 μM, SI = 1246.66) and exhibited a virucidal effect at 10 μM in Vero cells. Combining the extract (0.5 μg/mL) or dolastane (0.5 μM) with ribavirin (0.5 μM) inhibited 80 to 90% of ZIKV replication, indicating a synergistic effect. Both the extract and dolastane also showed anti-*Chikungunya virus* activity [152].

The dichloromethane extract of the red seaweed *Bryotamnion triquetrum* showed inhibitory activity on the ZIKV replication in Vero cells with values of CC_50_ = 400.00 ± 13.50 µg/mL, EC_50_ = 1.38 µg/mL and SI = 289.85. The investigation of its mechanism of action revealed a moderate virucidal effect and moderate inhibition at the attachment stage of the virus in the host cell [153].

Roseotoxin A and B, destruxin A, F, Ch1 and Br1, along with chlorohydrin, are cyclohexadepsipeptides obtained from the extract of the marine fungus *Beauveria felina* SX-6-22. These compounds, tested at 10 μM, were able to inhibit ZIKV total RNA replication and NS5 production levels in virus-infected A549 cells and showed low cytotoxicity, which was observed by qRT-PCR [150]. The authors reported that felinotoxin E, roseotoxin B, and roseotoxin A exert antiviral activity during the early stages of infection based on a time-course detection assay. Moreover, it was proposed that the cyclohexadepsipeptides may act in the endosomal fusion, according to an assay that tracks the organelles acidification [154].

Overall, 3 out of 23 indole alkaloids isolated from the marine fungi of the genus *Fusarium* cultivated on GPY medium supplemented with L-tryptophan exhibited significant inhibitory activity against ZIKV in A549 cells, being fusaindoterpene B (EC_50_ = 7.5 μM), JBIR-03 (EC_50_ = 4.2 μM) and 1,2-bis(1H-indol-3-yl)ethane-1,2-dione (EC_50_ = 5.0 μM) identified as the active constituents [155].

Four semisynthetic derivatives of dolabellane diterpenes from species of *Eunicea* soft corals showed antiviral activity against ZIKV in a plaque reduction assay using Vero cells. The derivatives (1*R*, 3Z, 7*R*, 8*R*, 11*S*)-7,8-epoxy-13-ketodolabell-3-en-16-al (CC_50_ = 750 μM, EC_50_ = 0.90 ± 0.08 μM, SI = 830), (1*R*, 7*R*, 8*R*, 11*S*, 13*S*)-7,8-epoxy-13-hydroxy-dolabella-3,12(18)-diene (CC_50_ = 580 μM, EC_50_ = 1.2 ± 0.1 μM, SI = 480), (1*R*, 3*R*, 4*R*, 7*R*, 8*R*, 11*S*, 13*S*)-3,4:7,8-diepoxy-13-hydroxy-dolabell12(18)-ene (CC_50_ = 1000 μM, EC_50_ = 8.90 ± 0.10 μM, SI = 110) and (1R*, 4*R*, 5*R*, 8*S*, 12*R*, 14*R*)-4-hydroxy-10-keto-1-methoxydolast-9-(17)ene (CC_50_ = 730 μM, EC_50_ = 1.80 ± 0.10 μM, SI = 410) exhibited high antiviral activity in a concentration-dependent manner [156].

#### 3.2.6. NPs from Animals

The peptide lycotoxin-An1a (Av-LCTX-An1a) was isolated from the venom of the spider *Alopecosa nagpag*. It inhibited ZIKV replication in HUVEC and A549 cells, at 10 μM, observed by a real-time fluorescence assay, and acted as a competitive inhibitor of the NS2B-NS3 protease of ZIKV with a K*i* value of 12.54 ± 1.88 μM determined by the method of Dixon. It was also able to inhibit DENV-2 infection. No SI value was reported in this study [157].

Quinoline has been so far reported as a natural product from the Peruvian stick insect *Oreophoetes peruana* [158]. Barbosa et al. (2017) [159] evaluated the activity of quinoline derivatives against ZIKV on Vero cells. Expressive activity against ZIKV was observed for the synthetic quinoline derivatives N1-(2,8-bis(trifluoromethyl)-quinolin-4-yl)ethane-1,2-diamine (CC_50_ = 195.00 ± 8.90 µM, EC_50_ = 0.80 ± 0.06 µM, SI = 243.75) and 2-[(2,8-bis(trifluoromethyl)quinolin-4-yl)amino]ethanol (CC_50_ = 189.00 ± 10.00 µM, EC_50_ = 0.80 ± 0.03 µM. SI = 236.25) showed activity against ZIKV-infected Vero cells five times higher than mefloquine (EC_50_ =3.6 ± 0.3 μM, CC_50_ = 212.0 ± 14.0 μM, SI = 58.9) [159].

The peptide Yodha, initially isolated from the skin of the frog specie *Indosylvirana aurantiaca*, and then obtained by synthesis, showed antiviral activity against different strains of ZIKV in Vero cells evaluated through qRT-PCR and focus-forming assay. The peptide showed no toxicity to human red blood cells up to 1000 μM. The cells treated with Yodha before the infection showed only 20% ZIKV RNA in comparison to the controls. In addition, we observed a decreased expression of the ZIKV envelope protein in samples treated with the Yodha peptide, according to the results of an immunofluorescence assay. Moreover, through electron microscopy, it was possible to observe that ZIKV treated with the Yodha peptide showed significant structural disruptions [160].

The peptide brevinin-2GHk (BR2GK) was isolated from the skin of the amphibian *Fejervarya limnocharis.* BR2GK reduced the virus-induced cytopathic effect in ZIKV-infected Vero cells and diminished the expression of ZIKV genomic RNA, E protein, and NS1. A time-addition assay was carried out and indicated that BR2GK showed significant inhibitory activity in the early and middle stages of ZIKV infection. In addition, a cell-based immunodetection assay revealed that BR2GK blocked ZIKV E protein expression with an EC_50_ of 3.40 ± 0.73 μΜ. BR2GK inactivated ZIKV by disrupting the integrity of the envelope and may also penetrate the host cell membrane to inhibit the middle stage of ZIKV infection [161].

#### 3.2.7. Miscellaneous NP

The retinoid N-(4-hydroxyphenyl) retinamide, present in foods of plant origin, inhibited Asian lineages of ZIKV in multiple mammalian cell lines including Vero (kidney, EC_90_ = 0.90 ± 0.09 µM), Huh7 (liver, EC_90_ = 1.00 ± 0.20 µM), BHK (kidney, EC_90_ = 0.90 ± 0.30 µM) and SK-N-SH (neuronal, EC_90_ = 1.00 ± 0.10 µM) cells, all of them with EC_90_ values near 1 µM. On the other hand, a less potent antiviral activity was observed in the mosquito cell line C6/36 (mosquito larvae, EC_90_ ≥ 20 µM), all of [162].

The antiviral peptide AH-D, obtained from a non-structural NS5A protein of *Hepatitis C virus*, destabilizes the viral lipid membrane. AH-D was active against ZIKV in Vero cells (CC_50_ = 63.4400 µM, EC_50_ = 0.0119 µM, SI = 5331.0924) [163].

A set of 24 nucleoside analogue ProTides, obtained by synthesis, was tested against ZIKV in Vero cell culture and afforded the nucleoside analogue 2′-C-methylcytidine as a promising anti-ZIKV compound (CC_50_ > 100 µM, EC_50_ = 0.30 ± 0.12, and SI > 333). The compound was tested in a luminescence-based cell survival assay in a human fetal neural stem cell model of ZIKV infection. It protected the cells from virus-induced cell death with an EC_50_ of 8.56 μM and a CC_50_ of >100 μM [164].

Thirteen synthetic trimers and tetramers of tryptophan were tested against ZIKV in Vero cells. Researchers observed anti-ZIKV activity for trimers AL439 (CC_50_ = 80.0 ± 30.0 µM, EC_50_ = 5.8 ± 1.1 µM, SI = 18.8) and AL440 (CC_50_ = 98.0 ± 3.0 µM, EC_50_ = 3.3 ± 1.4 µM, SI = 29.7). Compound AL439 was also active against ZIKV on Vero, BHK, Huh, Jeg3, A549, and HUVEC cell linages, with E_50_ values ranging from 0.6 to 5.8 µM. Additionally, compounds AL439 and AL440 showed antiviral effects against four serotypes of DENV [165].

### 3.3. NPs Tested in Animal Models of ZIKV Infection

A reduced number of NPs have been evaluated in laboratory animals infected with ZIKV. The reviewed literature indicates that so far, 12 compounds have been reported to induce anti-ZIKV activity in animal models, of which only 25-hydroxycholesterol was assayed in mice and in non-human primates (Table 2). Twelve compounds showed promising antiviral effect, reducing the viremia caused by ZIKV infection. However, the obtained results cannot be directly translated into humans [166].

Figure 2 shows the chemical structures of some selected NPs with potential anti-ZIKV activity discussed in this review. These compounds were selected based on their selectivity index values above 500 and/or because their potential antiviral effect has been demonstrated in more than one assay (in silico, in vitro or in vivo assays).

## 4. Discussion

The present revision covers the scientific literature on natural products tested against ZIKV published from January 1997 to December 2022. It comprises 157 original articles (Figure S1 in Supplementary Material), which is a reduced number of publications taking into account the impact of ZIKV infection on public health. This small number of articles demonstrates the slow progress on the research and development of bioactive NPs potentially useful as anti-ZIKV agents. In addition, it reinforces the current scenario that ZIKV infection is still a neglected tropical disease. In most countries, the epidemic outbreaks occurred in areas of low basic sanitation, which contributes to the proliferation of *Aedes* spp. and other ZIKV-transmitting mosquitoes [167].

The majority of studies herein reviewed—totalizing 82.4%—were carried out with natural products from plants and derivatives. The remaining publications report data obtained for NPs from fungi (3.1%), bacteria (7.6%), animals (1.2%), and marine organisms (1.9%) along with miscellaneous compounds (3.8%). Therefore, the vast majority of natural products so far investigated with anti-ZIKV activity were performed with plant secondary metabolites, mostly alkaloids, tannins, flavonoids, terpenoids, steroids, polyphenols, saponins, and stilbenes, among others. Phenolic compounds, especially flavonoids, comprise the largest number of natural products reported to possess anti-ZIKV activity.

The reported NPs mostly act at the stages of viral adsorption and internalization, and they present a virucidal effect. The reviewed data demonstrate their potential for developing new anti-ZIKV agents and also highlight the importance of additional studies addressing the molecular mechanisms of action of promising compounds such as epigallocatechin gallate (SI ≥ 25,000) [101], anisomycin (SI ≥ 11,900) [145], dolastane (SI = 1246) [153], and myricetin (SI ≥ 862) [101], among others. Some of the active compounds against Zika virus have been shown by in silico and in vitro assays to be active against other clinically important viruses and are promising candidates for further investigations aiming to develop broad-spectrum antivirus agents.

The chemical diversity of NPs offers the possibility of targeting different stages of the ZIKV infection and replication processes. For example, epigallocatechin-3-gallate and resveratrol show a virucidal effect and inhibit ZIKV adsorption to the host cell [114,122], whereas digitonin and conessine act on the stages of viral adsorption and internalization [116]. Furthermore, natural products tend to have more favorable physicochemical properties and lower cytotoxicity than synthetic compounds [110]. On the other hand, the intensive and time-consuming processes required for the extraction and isolation of NPs may limit or retard their application in drug development [168].

**Figure 3:**
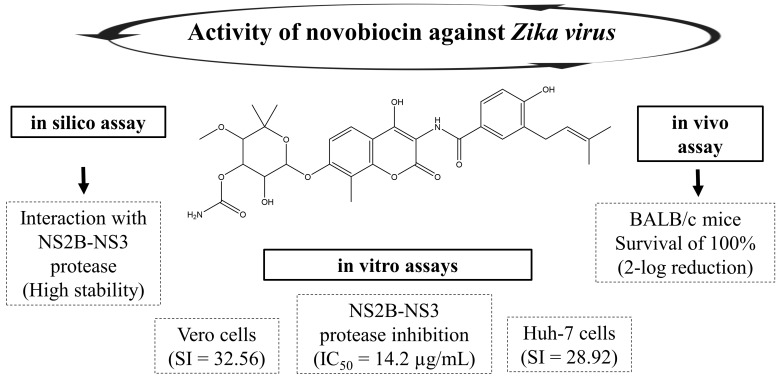
Nobobiocin, isolated from *Streptomyces niveus*, is an anti-ZIKV compound, whose activity has been predicted by in silico data and demonstrated by in vitro and in vivo assays. Median inhibitory concentration (IC_50_), selectivity index (SI), and in vivo data from [43].

It should be remembered that the prior evaluation of activity prediction of natural products by in silico approaches represents a “starting point” for further in vitro and in vivo testing. The coumarin-derived antibiotic novobiocin obtained from *Streptomyces niveus* is an example of an NP that presented promising results on in silico studies, which were further confirmed by results from in vitro and in vivo assays (Figure 3).

The knowledge about the antiviral activity of flavones dates to the 1990s, when it was demonstrated that the simultaneous administration of apigenin with acyclovir amplified the antiviral effect in vitro against *Herpes virus* types 1 and 2 (HSV-1 and HSV-2) [169]. Moreover, the antiviral activity of different flavonoids has been described by using in vitro and in vivo models, such as apigenin against adenovirus and hepatitis B, baicalein and isorhamnetin against *Influenza virus*, and chrysosplenol C against poliovirus type 3, among others, to cite a few examples [169,170]. Another polyphenol, epigallocatechin-3-gallate, has been reported to exert a broad spectrum of action against several viruses such as Hepatitis B virus (HBV), HSV, Epstein Barr virus (EBV), Adenovirus, Human Immunodeficiency virus (HIV), Hepatitis C virus (HCV), Influenza virus, DENV, Japanese encephalitis virus (JEV), Borne encephalitis virus (TBEV), CHIKV, and also ZIKV, among others [171]. According to the authors, epigallocatechin-3-gallate can be regarded as a nucleophilic reagent, since the phenolic hydroxyl groups found in the pyrogallol and galloyl moieties provide a higher number of lone electron pairs than other catechins. Therefore, it can react or combine with different molecules under appropriate conditions, thus inducing a broad antiviral effect.

According to Newman and Cragg (2020) [23], NPs still offer the best strategy for finding new bioactive compounds that may lead to effective agents for treating a variety of human diseases, including antiviral candidates. Different strategies have been adopted for this purpose, including the repositioning of drugs (e.g., mefloquine, hydroxychloroquine, chloroquine, and silymarin) and the screening of libraries of bioactive NPs and derivatives. Among these, chloroquine stands out as a promising compound based on the results from in vitro and in vivo tests. In addition, chloroquine has a defined mechanism of action, as it blocks the virus internalization in the host cell, which may favor its development as an antiviral drug. It can be therefore expected that one of the active NPs might be further investigated and developed as a drug or an herbal preparation for preventing or treating ZIKV infection. However, there is limited progress in this direction, and only one preclinical study has been so far reported in non-human primates [129]. To the best of our knowledge, there is no report of a clinical trial conducted with an NP or derivatives.

Another point to highlight is that several NPs herein reviewed have been reported to exert antiviral activity against ZIKV and to several other viruses such as DENV (genus *Flavivirus* and family *Flaviviridae*), West Nile virus (genus *Flavivirus* and family *Flaviviridae*), HCV (genus *Hepacivirus* and family *Flaviviridae*), HSV (genus *Simplexvirus* and family *Herpesviridae*), HIV (genus *Lentiviru*s and family *Retroviridae*), Human cytomegalovirus (genus *Cytomegalovirus* and family *Herpesviridae*), JEV (genus *Flavivirus* and family *Flaviviridae*) and CHIKV (genus *Alphavirus* and family *Togaviridae*), including anisomycin [145], actephilol B3, actephilol C, actephilol A, epi-actephilol A, fimbricalyx C, and fimbricalyx D [109], nanchangmicin [140], labyrinthopeptins A1 [143], cavinafungin [148], brefeldin A [149], and lycotoxin-An1a [157], along with AL439 and AL440, which are derivatives of tryptophan [165]. Members of the *Flaviviridae* family share similarities in their morphology, genomic organization, and replication strategies [172]; these findings open perspectives for developing broad-spectrum antivirals. Therefore, NPs and derivatives are promising sources of potential anti-ZIKV or anti-*Flavivirus* agents.

## 5. Conclusions

The vast majority of the studies performed to investigate the anti-ZIKV effect of extracts, natural products, and derivatives were carried out using in vitro assays. The ethanolic extract of the seaweed *Osmundaria obtusiloba*, along with the aqueous extracts of aerial parts from the plants *Psiloxylon mauritianum* and *Aphloia theiformis*, were identified as the most active ones. In its turn, epigallocatechin gallate, anisomycin, dolastane, and myricetin were disclosed as the most promising compounds for developing new anti-ZIKV agents based on results from in vitro and in vivo assays. Of note, the number of investigations so far performed using animal models is limited, especially using non-human primates. Therefore, it is mandatory to prioritize the pre-clinical investigation of the promising compounds disclosed by in vitro assays, aiming to select candidates for clinical studies. To the best of our knowledge, no data on the clinical investigation of NPs or derivatives with an anti-ZIKV effect have been so far reported. The development of antiviral agents that act both in prophylaxis and the treatment of ZIKV infection is urgently demanded, taking into account the severe outcomes of ZIVK infections to the population of several countries, especially to individuals living in low sanitation areas. Furthermore, a global effort is needed to control not only ZIKV but also other endemic species of *Flavivirus* and possible future outbreaks. Importantly, compounds showing high selectivity for ZIKV are considered potentially active against other members of the *Flavivirus* family. Therefore, the identification of bioactive NPs and derivatives and their mechanisms of extra- and intra-cellular action constitute a suitable strategy for the development of new antiviral drugs.

## Figures and Tables

**Table 2:** Effect of NPs and derivatives on *Zika* virus-infected animals.

Compound	Source	Animal Model/Dose	Outcomes on ZIKV Infection/[Reference]
*N*-(4-hydroxyphenyl) retinamide, fenretinide	Food of plant origin	AG129 mice, 60 mg/kg/2×/day, i.p. every 12 h from 24 h pre-infection to 84 h post-infection	Significant reduction in serum viremia, viral load in the brain, and viral RNA synthesis without direct inhibition of viral polymerase or chemical destabilization of membrane-associated replication complexes [162]
Novobiocin	*Streptomyces* sp.	BALB/c mice, 100 mg/kg/2×/day, s.i., treatment until 13 dpi	Survival of 100% novobiocin-treated mice vs. 0% untreated control mice. The mean viral loads of the blood and most major organ tissues of the novobiocin-treated mice were significantly lower than those of the untreated control mice (with ≥2-log reduction) at both 5 dpi and 14 dpi [43]
25-hydroxy-cholesterol (25HC)	Food of animal origin	BALB/c mice, 50 mg/kg, i.p., one dpi treatment A129 mice, 50 mg/kg, i.p., daily treatment for 7 days *Rhesus* monkeys, 1.5 mg/kg/day, i.v., intravenously, daily treatment for 7 days	BALB/c mice: one dpi treatment reduced viremia. A129 mice: reduced mortality significantly, and the surviving mice developed no clinical symptoms. The ZIKV titer in the brains of 25HC-treated animals was reduced at 7 dpi; Monkeys: treatment reduced viremia and inhibited ZIKV infection; the animals did not develop fever in response to infection [129]
Quercetin-3-β-*O*-D-glucoside	Fruits, vegetables, leaves and grains	*Ifnar1*^−/−^ mice, 50 mg/kg, one dpi treatment	50% of treated animals survived the infection with an average weight loss of 20% [121]
Chloroquine	Derivative of quinine (isolated from *Cinchona pubescens*)	BALB/c and A129 mice, 100 mg/kg, i.g., 6 h before ZIKV infection and once daily for the following 5 days	Treatment attenuated ZIKV-associated morbidity and mortality, protected fetal mice from microcephaly, and reduced viremia in adult mice [89]
27-mer amphipathic, α-helical d-enantiomer (AH-D)	Non-structural NS5A protein of *Hepatitis C virus*	IFN-α/βR^−/−^ SV129 (A129^−/−^) mice, 25 mg/kg/2×/day, i.p.	Treatment reduced viral loads (3-log), prevented brain damage, reduced clinical symptoms, diminished lethality and preserved the integrity of the blood–brain barrier. Treatment reduced viral loads in serum, spleen, brain, and optical nerve [163]
Anisomycin	*Streptomyces griseolus*	AG129 mice, 4 mg/kg/day, s.i., for 10 days starting 4 h post-infection	Treatment reduced viremia levels and prologued the survival rates [145]
Lycorine	*Crinum jagus*	AG6 mice, 1 mg/kg, 5 mg/kg and 10 mg/kg/day, i.g., intragastric administration 14 days	Treatment resulted in survival rates of 33%, 66%, and 82%, respectively, for the groups treated with 1, 5, and 10 mg/kg. Treatment with the highest dose reduced RNA viral in the liver and brain tissue as well as presented less pathological damage in comparison to the other doses [63]
Cinnamic acid	Cinnamon oil	AG6 mice, 150 mg/kg/day, i.g., 12 days treatment	Treatment reduced mortality by 80%; decreased the viral RNA copy number in the mice serum and ZIKV protein content in the mice brain [107]
Ascomycin	*Streptomyces hygroscopicus*	A129 mice, 1 mg/kg/day, i.p. 5 days treatment	Reduced ZIKV load was observed 48 h post-treatment in blood and 120 h post-treatment in brain. According to immuno-histochemical analysis, the treatment significantly reduced the ZIKV E protein positive cells in the cortex, hippocampus, and thalamus of mice brains [146]
Yodha	Amphibian, e.g., frog skin	C57BL/6 mice, 0.1 mg/kg/day, i.p., 3 days treatment	Treatment reduced viremia and ZIKV viral load in eyes and spleen [160]
Rottlerin	Camara powder	Kunming neonatal mice, 6 mg/kg, intracerebral injection, 14 days treatment	At 10 dpi, rottlerin treatment reduced the weight loss and severity of hind limb paralysis caused by ZIKV infection. The viral RNA copies and viral loads in the brains of rottlerin-treated mice were dramatically lower than the control mice. At 14 dpi, 75% of the rottlerin-treated mice survived [134]

i.g. = intragastric administration; i.p. = intraperitoneal injection; i.v. = intravenous injection; s.i. = subcutaneous injection; dpi = days post-infection.

## Data Availability

No new data were created or analyzed in this study. Data sharing is not applicable to this article.

## Supplementary Materials

The following supporting information can be downloaded at: https://www.mdpi.com/article/10.3390/v15051211/s1, Figure S1: Number of scientific articles reporting natural products tested against ZIKV, published between January 1997 and December 2022. Total of 157 articles; Table S1. Natural products that interact with molecular targets of ZIKV identified by in silico assay; Table S2: Anti-ZIKV activity induced by natural products and derivatives, assayed by in vitro methods that showed selectivity index (SI) ≤ 10. References [84,173,174,175,176,177,178,179,180,181] are cited in the supplementary materials.
