# Supplementary material for: Natural Products and Derivatives as Potential Zika virus Inhibitors: A Comprehensive Review
Source: Viruses. 2023 May 20;15(5):1211. doi: 10.3390/v15051211 (PMC10222392; doi:10.3390/v15051211)
Supplement: Supplementary file 1 [file viruses-15-01211-s001.zip › viruses-2299165-supplementary.pdf]

SUPPLEMENTARY MATERIAL

Natural Products and Derivatives as Potential *Zika Virus* Inhibitors: A Comprehensive Review

Rosângela Santos Pereira [1], Françoise Camila Pereira Santos [1], Priscilla Rodrigues Valadares Campana [1], Vivian Vasconcelos Costa [2], Rodrigo Maia de Pádua [1], Daniele da Glória de Souza [3], Mauro Martins Teixeira [2] and Fernão Castro Braga [1,*]

[1] Department of Pharmaceutical Products, Faculty of Pharmacy, Universidade Federal de Minas Gerais, Belo Horizonte 31270-901, MG, Brazil; rosa.rspereira@gmail.com (R.S.P.)

[2] Department of Biochemistry and Immunology, Institute of Biological Sciences, Universidade Federal de Minas Gerais, Belo Horizonte 31270-901, MG, Brazil

[3] Department of Microbiology, Institute of Biological Sciences, Universidade Federal de Minas Gerais, Belo Horizonte 31270-901, MG, Brazil

* Correspondence: fernaobraga@farmacia.ufmg.br

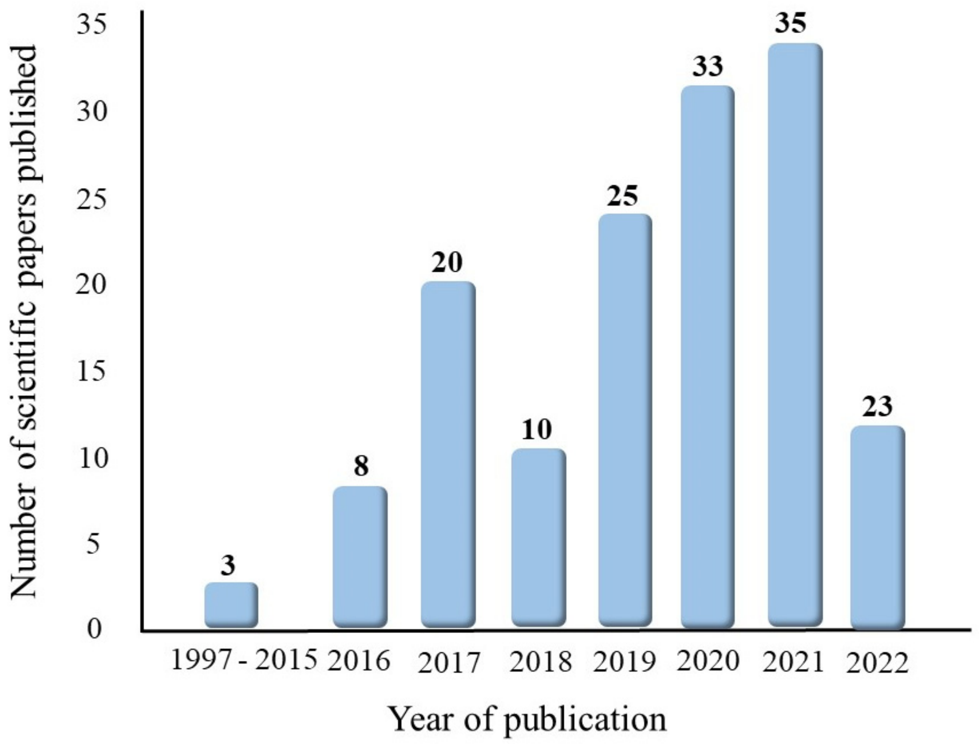

Figure S1: Number of scientific articles reporting natural products tested against ZIKV, published between January 1997 and December 2022. Total of 157 articles.

Table S1. Natural products that interact with molecular targets of ZIKV according to *in silico* data.

***Zika virus* molecular target**	**Natural products that interact with the target**
MTase domain of NS5	Quercetagetin, Chrysophanic Acid, Jaceidin, Methylglovanon, Caninnot, Luteolin, Quercetin, Redentin, Anthranol, Heliannone B, Hydroxy Kemferol, Secterpathe and Beta Caryophylene [34]
	Dihydroxy-348-trimethoxy, Isopomiferin, Silydiamin B, Amyrisin C, Cannflavin, Derrisin, Mundinol, Schizolaeone B, Tomentodiplacone G, Silydianin, Diprenyleriodictyol, Isosilybin A, Silydin D, Taxifolin,, Silybin C, Silybin B, Silybin A, Lupiwighteone, Euchrenone B, Eryvarin Q, Eryvarin O, Abyssinone V, Sigmoidin C, Sigmoidin B, Robustone, Erycristagallin, (+)-Syringaresinol, Panthenolide, Papraline, Lycopene, Paprarine, Papraine, Isoach, Fumaritine-N-oxide and Narlumirine [44]
RdRp domain of NS5	Ferulic Acid, 1-Aminocyclopropane-1-carboxylic acid, Cinnamic Acid, Bornylacetate, Salicylic Acid, Cymene, Gamma, Kolin, Fernesene, Myrtenal, Alphaprodine, Beta Pinene, Xylene, Benzaldehyde, Nicotinic Acid, Tridecanoic and Cyclopentadiene [34]
	Amyrisn A, Isopomiferin, Silydianin B, Isomangostin, Mearnsetin, Cannflavin, Derrisin, Mundinol, Schizolaeone B, Tomatidiplacone G, Anthraxin, Diprenyleriodictyol, Isosilybin B, Isosilybin A, Isoerysenegalenseine, Euchrenone B, Eryvarinols A, AbyssinoneV, Sigmoidin C, Sigmoidin B, Robustone, Oxyresveratrol, Osajin, Erycristagallin, Paprarine, Estafin, FumaritineN-oxide, Papracinine and Oxysanguinarine [44]
	Lycorine [63]
	Xanthoangelol [46]
	Polydatin, Dihydrogenistin, Liquiritin, Rhapontin and Cichoriin [48]
Envelope protein domain III	Bacopaside III and bacoside A [51]
	Epicatechin, baicalin, isonimolicinolide, madecassic acid, and apigenin-7-*O*-beta-D-glucopyranoside [39]
	4-Hydroxy-2-methylacetophenone, Stigmasterol, 6-Octen-1-ol,3,7-dimethyl, megastigmatrienone, myclohexylmethyl hexyl ester and testerone cypionate [55]
	Palmatine [41]
	Epigallocatechin gallate (EGCG) [53]

	Tanic acid [52]
	Harringtonine [42]
	Chicoric acid, luteone, reserpine, and rosmarinic acid [60]
	Pentagalloylglucose, Parishin A and Stevioside [54]
	Saikosaponin D; Cimiside F; Pomolic acid; Esculentoside A; Esculentoside B; Tinoside5,7,2',5'-Tetrahydroxy-flavone; Andrographatosid; Cimicifugic acid B1; Cyanidin3,5-diglucoside; Bruceine F; Delphinidin; Arnicolide D and Kirenol [56]
Capsid protein	Chicoric acid, luteone, reserpine, and rosmarinic acid [60]
Protease	Saikosaponin D; Cimiside F; Methyl euscaphate; Ganoderic acid Y; Pomolic acid; Macrophylloside D; Mudanpioside E; -Chrysanthemin; 8-epi-Loganic acid; Cyanidin 3,5-diglucoside; Arnicolide D; Delphinidin; 6,7-Dehydroartemisinic acid and Cylindrene [56]
Polymerase	Saikosaponin D; Cimiside F; Esculentoside A; Ganoderic acid Y; Methyl euscaphate; Macrophylloside D; Dihydrobrusatol 1; Tinoside; Andrographatoside; Cyanidin3,5-diglucoside; Delphinidin; Bruceine F; Schizonepetoside A and Nigakilactone K [56]
NS1 protein	Flavonoid-rich fractions (F1 to F10) [60]
	Tangeretin [61]
NS1 e NS3 protein	Bacopaside III and bacoside A [51]
NS2B/NS3, serine protease	Hesperetin (HST) [35]
Helicase	Saikosaponin D; Esculentoside A; Cimiside F; Esculentoside B; Pomolic acid; Benzoyloxypaeniflorin; Macrophylloside D; Chrysanthemin; Mudanpioside E; Tinoside; Cyanidin 3,5-diglucoside; Bruceine F; Delphinidin; Schizonepetoside A and Retinol [56]
NS3 helicase protein	Cassiarina D, 3'-O-metildiplacona, exiguaflavanona A e lactucopicrina [28]
	Baicalein, catechin, muricatetrocin, canthin, eleutheroside B, ellagic acid, epigallocatechin, tneoandrographolide, ponapensin, sangennon [39]
	5,3’-dihydroxy-3,6,7,8,4′-pentamethoxyflavone (5DP); 5-hydroxy-3,6,7,8,3′,4′-hexamethoxyflavone (5HH); myricetin-3-O-rhamnoside (M3OR) [50]
	Rutin, Nicotiflorin, Isoquercitrin, and Hyperoside [66]
NS2B-NS3 protease	Quecertin [32]
	Flinderol A, flinderol B, angusticornin B, curaridine [28]
	Bromocriptine [29]
	Novobiocin [57]
	Myricetin; Quercetin; Luteolin; Isorhamnetin; Apigenin, Curcumin [31]

	Hydroxychloroquine [30]
	Narigenin [36]
	Quercetin, rutin and pedalitin [38]
	Epigallocatechin gallate, epigallocatechin gallate-7-*O*-glucopyranoside, epigallocatechin gallate-4'-*O*-α-glucopyranoside, isoquercetin, rutin, sanggenon O [40]
	Berberine derivatives (4a, 4b, 4c, 4d, 5a, 5b, 5c and 5d) [43]
	Glycyrrhetinic acid derivatives 13 and 19 [62]
	Bisandrographolide, Andrographolide, and Andrographiside [45]
	Chicoric acid, luteone, reserpine, and rosmarinic acid [60]
	β-caryophyllene [49]
	Bixin, annatto, crocetin dimethyl ester, ethyl bixin, mycorradicin, norbixin and transcrocetin [47]
NS5 methyltransferase	Cimicifenol, cimiracemato B, ácido rosmarínico, kanzonol Y, curaridina, kanzonol V, solofenol D, (-)-asarinina [28]
	Glycyrrhizic acid derivatives conjugated with amino acid (Compound 13 e 14) [62]
	Theaflavin [65]
	Bixin, annatto, crocetin dimethyl ester, ethyl bixin, mycorradicin, norbixin and transcrocetin [47]
NS5 protein	Baicalein and baicalin [37]
	β-caryophyllene [49]
	Tangeretin [61]
NS5 RNA-dependent RNA polymerase	4',7-Digalloylcatechin-di-*O*-dimethylisoguaiacin, 2,4,4'-trihydroxy-3,3'-diprenylchalcone, flinderol B [28]
	Piperine and Isoscutellarein [64]
	Chicoric acid, luteone, reserpine, and rosmarinic acid [60]
	Rutin, Nicotiflorin, Isoquercitrin, and Hyperoside [66]
Methyltransferase	Sinefungin [58]
	Saikosaponin D; Esculentoside B; Ganoderic acid Y; Methyl euscaphate; Cimiside F; Benzoyloxypaeniflorin; Chrysanthemin; Cimicifugic acid B1; Macrophylloside D; 5,7,2',5'-Tetrahydroxy-flavone; Mudanpioside E; Cyanidin 3,5-diglucoside; Retinol; Bruceine F; Delphinidin and Schizonepetoside A [56]
TNF	Curcumin [33]

Table S2. Anti-ZIKV activity induced by natural products and derivatives, assayed by *in vitro* methods, that showed selectivity index (SI) ≤ 10.

**Source**	**Sample**	**Anti-ZIKV activity**		**Reference**
		Cell linage or Assay model	SI / Biological effect	
Derivatives of quinine	chloroquine	hBMECs cells	SI = 8.21	[90]
Derivatives of quinine	chloroquine	neural stem cells	SI = 7.68	[90]
Bacterium *Streptomyces narbonensis*	lovastatin	Huh7 cells	SI > 2.5	[173]
Fungus *Aspergillus terreus*	kitasamycin,	Huh7 cells	SI = > 1.2	[173]
-	(+)-*trans*-dihydronarciclasine SC, narciclasine SC and pancratistatin SC	Vero C-1008 cells	SI = 7; 4 and 4 respectively	[174]
-	Bromocriptine PC	ZIKV NS2B-NS3 protease inhibition assay<br><br>Vero cells	IC_{50} = 21.6±1.1µM<br><br>SI > 3.07<br>Interferes with the steps after the internalization of the ZIKV	[29]
Leaves (L) and Branches (B)<br><br>*(Tontelea micranta)*	Hexane EX (L), ethyl acetate (L), methanolic (L), hexane EX (B), chloroform (B) and ethyl acetate (B)	Vero cells	SI = 2.66; 3.03; 4.23; 4.70; 9.60 and 2.85<br>Virucidal effect, strongly acting on the viral particle, and inhibited the infection at the adsorption and penetration stages, except for the hexane branch extract.	[175]
Tecoma castaneifolia Trunks and leaves	Ethanolic, EX	Vero cells	SI = 1.53<br>SI > 3.27	[176]
Tecoma garrocha Trunks and leaves	Ethanolic, EX	Vero cells	SI= 1.21<br>SI > 1.33	[176]
Tecoma stans var. *stans* Leaves	Ethanolic, EX	Vero cells	SI> 2.03	[176]
Tecoma stans var. *angustata*	Ethanolic, EX	Vero cells	SI > 3.73	[176]

Table S2. *Cont.*

Leaves				
Tecoma stans var. *stans* Trunks	Ethyl acetate EX, FR; Aqueous FR and Crenatoside IC	Vero cells	SI = 1.33; SI = 2,53 and SI = 4,25	[176]
Cissus erosa Stems and leaves	Stems ethanolic EX and Leaves ethanolic EX	Vero cells	SI = 8.5 and 3.7 respectively	[177]
Ehretia microphylla; Combretum indicum; Psidium guajava; Clinopodium douglasii; Blumea balsamifera; Peperomia pelúcida; Vitex negundo; Momordica charantia; Senna alata and Allium sativum	Aqueous EX	Vero cells	SI = 1.2548; 1.0752; 2.1905; 1.0792; 1.6972; 0.2000; 1.8290; 2.2739; 1.0933 and 1.4160 respectively The authors suggest that the mechanism of viral inhibition is more likely to be via binding with ZIKV viral replication proteins	[56]
Derivatives of flavanone naringenin	7-(hexyloxy)-5-hydroxy-2-(4-hydroxyphenyl)chroman-4-one; 5-hydroxy-2-(4-hydroxyphenyl)-7-(octyloxy)chroman-4-one; 5-hydroxy-2-(4-hydroxyphenyl)-7-(nonyloxy)chroman-4-one and 7-(dodecyloxy)-5-hydroxy-2-(4-hydroxyphenyl)chroman-4-one All compunds are SC	Table S2. *Cont*. A549 cells	SI = 4.39; 4.12; 4.11 and 4.2 respectively	[178]
Isodon xerophilus Aerial parts	Enanderinanin J IC	A549 cells	SI = 5.6 The autophagy inhibitory activity of enanderinanin J protects host cells from these RNA viruses.	[179]
Ouratea semiserrata Stems	Ethanolic EX, epicatechin PC ,	Vero cells	SI = 1.30, >3.55, > 10.00 and 4.00 respectively	[180]

	catechin PC and rutin PC			
Bruguiera gymnorhiza Fruits and roots	Aqueous EX by maceration	A549 cells	SI = 3.0 and 3.3 respectively Inhibited the infection at the adsorption stage.	[181]
-	Berberine derivatives (4a, 4b, 4c, 5a,5b,5c and 5d)	Vero cells	SI = 4.1, 3.2, 4.7, 7.5, 2.4, 2.6, 2.8 and 2.7 respectively	[43]
-	Lycorine; Pretazettine; Narciclasine; Narciclasine-4-O-β-D-xylopyranoside and 1-acetyl-lycorine	Vero cells<br>Table S2. *Cont*.	SI = 3.8; 3.8; 6.0; 6.5 and 1.0 respectively	[182]
Angelica keiskei	Xanthoangelol-E	Vero cells	SI = 5	[46]
M. ilicifolia and *T. phaeocarpa*	-	SH-SY5Y cells	SI=3.4 and SI=4.8 respectively	[83]
Artemisia capilaris and *Hedyotis diffusa*	Aqueous EX	Vero cells	SI=5.2 and SI=4.9 respectively	[85]
-	Pedalitin and quercetin	Vero cells	SI=4.3 and SI=2.0 respectively	[123]

EC= extract; IC= isolated compound; FR = fraction; CC = Cytotoxic concentration; IC = Inhibitory concentration; EC= effective concentration; SI = selectively index; PC = purchased compound; SC = synthesized compound; nd = not determined. Vero = kidney epithelial cells of African green monkey; A549 = Human epithelial cells; hBMECs = human brain microvascular endothelial cells

References (not cited in the manuscript)

173. Pascoalino, B.S.; Courtemanche, G.; Cordeiro, M.T.; Gil, L.H.; Freitas-Junior, L. Zika antiviral chemotherapy: identification of drugs and promising starting points for drug discovery from an FDA-approved library. *F1000Res.* **2016**, *5*, 2523. doi: 10.12688/f1000research.9648.1
174. Revu, O.; Zepeda-Velázquez, C.; NIelsen, A.J.; Mcnulty, J.; Yolken, R.H..; Jones-Brando, L. Total synthesis of the natural product (+)-*trans*-Dihydronarciclasine via an asymmetric organocatalytic [3+3]-cycloaddition and discovery of its potent anti-*Zika virus* (ZIKV) activity. *ChemistrySelect* **2016**, *1*, 5895-5899. doi: 10.1002/slct.201601536
175. Ferreira, F.L.; Hauck, M.S.; Duarte, L.P.; Magalhães, J.C.; Silva, L.S.M.; Pimenta, L.P.S.; Lopes, J.C.D.; Mercadante-Simões, M.O.; Vieira Filho, S.A. *Zika virus* activity of the leaf and branch extracts of *Tontelea micrantha* and its hexane extracts phytochemical study. *J. Braz. Chem. Soc.* **2019**, *30*, 793-803. doi: 10.21577/0103-5053.20180210

176. Reis, A.C.C.; Silva, B.M.; de Moura, H.M.M.; Pereira, G.R.; Brandão, G.C. Anti-*Zika virus* activity and chemical characterization by ultra-high performance liquid chromatography (UPLC-DAD-UV-MS) of ethanol extracts in *Tecoma* species. *BMC Complement. Med. Ther.* **2020**, *20*, 246. doi: 10.1186/s12906-020-03040-0
177. Reis, A.C.C.; Moura, H.M.M.; Silva, B.M.; Oliveira, A.B.; Brandão, G.C. Antiviral activity and chemical characterization of *Cissus erosa* (Vitaceae) ethanol extracts. *Rodriguésia* **2020b**, 71. doi: https://doi.org/10.1590/2175-7860202071052
178. Mendes, L.A.O.; Ponciano, C.S.; Depieri Cataneo, A.H.; Wowk, P.F.; Bordignon, J.; Silva, H.; Vieira de Almeida, M.; Ávila, E.P. The anti-*Zika virus* and anti-tumoral activity of the citrus flavanone lipophilic naringenin-based compounds. *Chem Biol Interact.* **2020**, *331*, 109218. doi: 10.1016/j.cbi.2020.109218
179. Huang, L.; Fu, Q.; Dai, J.M.; Yan, B.C.; Wang, D.; Puno, P.T.; Yue, J. High-content screening of diterpenoids from *Isodon* species as autophagy modulators and the functional study of their antiviral activities. *Cell Biol. Toxicol.* **2021**, *37*, 695-713. doi: 10.1007/s10565-021-09580-6
180. Ferreira, G.M.; de Mello Silva, B.; de Souza, G.H.B.; de Oliveira, A.B.; Brandão, G.C. Anti-Zika activity of *Ouratea semiserrata* and dereplication of its constituents. *Rev. Bras. Farmacogn.* **2021**, *31*, 121-125. doi: 10.1007/s43450-021-00129-6
181. Bibi Sadeer, N.; Haddad, J.G.; Oday Ezzat, M.; Desprès, P.; Abdallah, H.H.; Zengin, G.; Uysal, A.; El Kalamouni, C.; Gallo, M.; Montesano, D.; Mahomoodally, M.F. *Bruguiera gymnorhiza* (L.) Lam. at the forefront of pharma to confront Zika virus and microbial infections-an *in vitro* and *in silico* perspective. *Molecules* **2021**, *26*, 5768. doi: 10.3390/molecules26195768
182. de Castro Barbosa, E.; Alves, T.M.A.; Kohlhoff, M.; Jangola, S.T.G.; Pires, D.E.V.; Figueiredo, A.C.C.; Alves, É.A.R.; Calzavara-Silva, C.E.; Sobral, M.; Kroon, E.G.; Rosa, L.H.; Zani, C.L.; de Oliveira, J.G. Searching for plant-derived antivirals against *Dengue virus* and *Zika virus*. *Virol. J.* **2022**, *19*, 31. doi: 10.1186/s12985-022-01751-z
